# LEAF: benchmarking multiobjective evolutionary feature selection over large language model–extracted symptoms for interpretable Traditional Chinese Medicine respiratory syndrome differentiation

**DOI:** 10.3389/fmed.2026.1990580

**Published:** 2026-10-28

**Authors:** Xuanhang Luo, Huan Ding, Xize Zhang, Binhuan Miao, Li Shi

**Affiliations:** 1 Changchun University of Chinese Medicine, Changchun, China; 2 Department of Pulmonology, Affiliated Hospital of Changchun University of Chinese Medicine, Changchun, China

**Keywords:** evolutionary computation, large language models, multiobjective feature selection, respiratory diseases, syndrome differentiation, Traditional Chinese Medicine

## Abstract

Differentiating Traditional Chinese Medicine (TCM) respiratory syndromes requires weighing overlapping symptoms together with tongue and pulse findings, a task that most AI systems for TCM perform end-to-end but without an interpretable intermediate representation. In this study, we addressed this gap with LEAF (LLM extraction and multiobjective feature selection), a two-stage modular framework evaluated on 3,253 records covering 14 respiratory syndromes from the TCM-SD benchmark. Its first large language model (LLM) stage extracted a 35-item symptom ontology that was built entirely from public TCM sources; among three interchangeable LLMs, Qwen2.5-14B-Instruct was ranked as the best, followed by Phi-4 and the TCM domain model BianCang. Its second evolutionary-computation stage benchmarked four multiobjective evolutionary algorithms [non-dominated sorting genetic algorithm-II (NSGA-II), strength Pareto evolutionary algorithm 2 (SPEA2), competitive mechanism-based multiobjective particle swarm optimizer (CMOPSO), and non-dominated sorting differential evolution (NSDE)] over 10 seeds, and they were compared using Friedman’s and Nemenyi’s testing in terms of both classification outcome and search-process quality. The four algorithms showed no significant separation on held-out macro-F1, although NSDE led nominally, and none exceeded the full-feature baseline on point estimates. In contrast, hypervolume differed significantly and favored NSGA-II, the weakest of the four on held-out macro-F1. A four-classifier panel found no algorithm to be significantly different from either the full-feature baseline or a random-feature control, which was significantly outperformed only by the full-feature baseline. Applying NSDE to the remaining two LLM pipelines reproduced the original ranking, and its representative solution was fully attested against the knowledge base of TCM-SD, although not typically so across all the 10 seeds. These results show that an LLM-extraction frontend built exclusively from public knowledge can provide a representation over which evolutionary feature selection cuts the candidate feature set roughly in half without a statistically detectable cost in performance, albeit at a small point-estimate cost; that hypervolume and inverted generational distance are not reliable stand-ins for classification-relevant algorithm selection here; and that a no-gold-standard proxy evaluation can substitute human annotation when expert time is limited. Because both stages plug in and out freely, the framework can absorb stronger components in either stage as they become available.

## 1 Introduction

Traditional Chinese Medicine (TCM) diagnoses respiratory diseases through syndrome differentiation, termed bianzheng: a practitioner integrates chief complaints, symptom history, tongue appearance, and pulse quality into one of a discrete set of syndrome patterns, each implying a distinct treatment principle. Respiratory syndromes are a particularly difficult case for this task: cough, dyspnea, and lung distension—known in Chinese as kesou, chuanzheng, and feizhang—share large, overlapping symptom vocabularies, and the diagnostic-relevant signal is distributed unevenly across free-text chief complaints, narrative history, and a templated four-diagnosis examination record, termed sizhen. Therefore, applying artificial intelligence (AI) to this task requires converting unstructured clinical text into a symptom representation before any classifier can be trained or interpreted.

Two gaps motivated us to conduct this study. First, the only large public benchmark for TCM syndrome differentiation (TCM-SD) provides free-text clinical records and syndrome labels but no structured symptom features, and it is not restricted to any single disease system; to our knowledge, no one has extracted a respiratory-specific, feature selection–ready subset from it. Second, existing TCM-AI work is predominantly end-to-end: a language model or a classifier consumes text and outputs a syndrome label directly without an interpretable intermediate representation for inspection by a clinician. Multiobjective evolutionary algorithms (MOEAs) are a known tool for producing such representations, as they can search for feature subsets that trade-off classification performance against parsimony and, potentially, consistency with documented clinical knowledge; however, doing so requires structured features as the input, which brings the pipeline back to the first gap.

In this study, we address both gaps with LEAF (large language model [LLM] extraction and multiobjective feature selection), which is a two-stage methodology. In the first stage, large language models (LLMs) were employed to extract a fixed, ontology-constrained set of symptom features from each record's free text via few-shot, schema-constrained prompting; information on tongue, pulse, and disease trigger is extracted separately by rule, since we found the four-diagnosis field to be dominated by a fixed physical examination template with little record-to-record variation outside its tongue/pulse tail. In the second stage, multiple MOEAs searched the resulting feature space for subsets that balance macro-F1 against feature parsimony, producing Pareto fronts of candidate symptom subsets in place of a single black-box decision.

A practical constraint shaped the methodology of the study as much as the technical design: the heavy clinical workload of the TCM practitioners involved in the project left no time for clinician to review the symptom ontology, to curate few-shot examples, or to annotate a symptom-level gold standard for evaluating the extraction quality. Instead of treating this as merely a limitation to disclose, we treat it as a design question: how far can a pipeline like this be built and validated using only public, static, peer-reviewed knowledge sources? We report the answer as part of the contribution. Concretely:

1. We constructed and released the methodology for a respiratory-disease subset of TCM-SD, comprising 3,253 records across 14 syndrome classes spanning eight disease categories after merging syndromes with fewer than 10 samples.
2. We built a 35-item symptom ontology with every term being traceable to the TCM-SD knowledge base, a published TCM expert-consensus guideline, or corpus keyword extraction, while a second guideline crossvalidates the syndrome taxonomy of the respiratory subset at the syndrome-name level, thereby substituting literature crossreferencing for clinician review. We further replaced symptom-level gold-standard annotation with a downstream proxy evaluation, a fixed-fold linear support vector machine (SVM) macro-F1 score, for screening candidate LLM extraction pipelines—a route we validated *post-hoc* by reproducing the same pipeline ranking after the full feature selection stage.
3. We deployed three LLMs locally—two general-purpose models, Qwen2.5-14B-Instruct and Phi-4, and one TCM domain–adapted model, BianCang–Qwen2.5-7B-Instruct—for schema-constrained structured symptom extraction and found that the domain-adapted model underperforms both general-purpose models on this task.
4. We compared four MOEAs spanning distinct search paradigms: non-dominated sorting genetic algorithm-II (NSGA-II), strength Pareto evolutionary algorithm 2 (SPEA2), competitive mechanism–based multiobjective particle swarm optimizer (CMOPSO), and non-dominated sorting differential evolution (NSDE) on the same continuous, threshold-decoded search-space representation and showed that the resulting feature subsets removed roughly half of the candidate symptoms without a statistically detectable loss in syndrome-differentiation performance relative to using all features.

Existing work applying artificial intelligence to TCM syndrome differentiation is predominantly end-to-end, and situating LEAF against it clarifies the second aforementioned gap. TCM-SD (1) remains the only large-scale public benchmark for TCM syndrome differentiation, and most subsequent work built on it, or on comparably scaled proprietary corpora, is end-to-end: a language model consumes free text and outputs a syndrome label directly with no interpretable structured layer in between. Yao et al. (2) fine-tuned Bidirectional Encoder Representations from Transformers (BERT) with continued pretraining on a TCM domain corpus and classified clinical records into five disease categories directly from character-level input, with no symptom-level feature engineering, reaching a macro-F1 of 88.64% on that coarser task. Liu et al. (3) combined a BERT-based entity representation with a conditional random field (CRF) tagger and an ensemble of deep text classifiers—a recurrent convolutional neural network (RCNN) and a hierarchical attention network (text-HAN)—for lung cancer syndrome differentiation, again mapping unstructured records to syndrome labels through model fusion instead of providing an inspectable intermediate representation. Gong et al. (4) is closer in spirit to our aim of interpretability: they proposed a multilabel deep forest classifier with an improved multilabel ReliefF feature selection step, termed PCC-multilabel ReliefF (MLRF), to handle small-sample, multisyndrome co-occurrence, showing that feature selection can improve TCM syndrome differentiation under label sparsity. However, their features are derived directly from structured inputs already present in their corpus, and the selection is single-objective ReliefF in place of a multiobjective evolutionary search over LLM-extracted symptom features. Wang et al. (5) took an orthogonal approach to structure: instead of mining features from a large weakly annotated corpus similar to how TCM-SD does, TCMEval-SDT expert-curates 300 cases annotated with a four-step diagnostic-thought chain (information extraction, pathogenesis reasoning, syndrome reasoning, and explanation), trading scale for annotation depth. None of these prior TCM-AI studies combines LLM-based structured symptom extraction with a multiobjective search over the resulting feature space, which is the gap LEAF addresses.

The extraction stage of LEAF builds on general-purpose instruction-tuned LLMs—Qwen2.5 (6) and Phi-4 separately—rather than relying solely on TCM-specific pretraining, and we evaluated these alongside a domain-adapted alternative. Domain adaptation has shown gains in TCM knowledge and diagnostic reasoning benchmarks. Yang et al. (7) continually pretrained a general LLM on a 1B-token TCM corpus with low-rank adaptation (LoRA) to produce TCM-GPT-7B, reporting relative accuracy gains of 17% and 12% over the compared models on TCM licensing exam and diagnosis tasks. BianCang (8)—the domain model that we evaluated directly—is built similarly through TCM-specific continued pretraining and instruction tuning. However, gains on knowledge recall and diagnostic reasoning benchmarks do not necessarily transfer to structured extraction tasks, which stress instruction-following and schema compliance more than TCM knowledge itself. Ouyang et al. (9) evaluated a broad set of Chinese medical LLMs, including domain-adapted models, on a 33,735-question clinical benchmark spanning 14 scenarios, and they found that Chinese medical domain models can underperform general-domain LLMs on reasoning and factual consistency dimensions, attributing part of the gap to the constrained input capacity that limits practical deployment. This pattern foreshadows our own extraction-screening result reported below, where the TCM domain model BianCang underperformed when compared with both the general-purpose models. Unlike the broad, multiscenario evaluation of CliMedBench, our screening isolates a single, narrow capability—schema-constrained structured extraction from free text—and we are not aware of any prior work comparing domain-adapted models against general-purpose LLMs specifically on this capability for TCM symptom extraction.

MOEAs for feature selection are a mature area (10); the four algorithms compared in this study span its major search paradigms. NSGA-II (11) established the fast non-dominated sorting and crowding-distance mechanism that dominance-based MOEAs still rely on, and SPEA2 refined its predecessor's archive-based elitism. NSDE instead applies mutation and crossover operators of differential evolution (12) within the non-dominated sorting framework of NSGA-II (11), representing the differential evolution–based paradigm among the four algorithms involved in this study. The competitive swarm mechanism of CMOPSO descends from the broader multiobjective particle swarm optimization (PSO) lineage surveyed by Reyes-Sierra and Coello Coello (13).

This area remains active in recent years. Asilian Bidgoli and Rahnamayan (14) proposed a multiphase search space–shrinking approach that applies a ranking-based filter preshrinking step before the evolutionary search itself, targeting large-scale, high-dimensional multiobjective feature selection. On the swarm-based paradigm of CMOPSO, Zhang et al. (15) proposed a self-adaptive multiobjective PSO, termed MOSaPSO, combined with a fast non–dominated sorting approach specifically for large-scale feature selection, reporting improvements over prior MOPSO variants across multiple classifiers. On the differential evolution paradigm of NSDE, Zhang et al. (16) proposed a multiobjective differential evolution method with a four subpopulation initialization strategy driven by feature weights and redundancy indices, which was evaluated against several state-of-the-art multiobjective feature selection methods on 11 datasets—a direct 2025 counterpart to the differential evolution–based search paradigm we compare with via NSDE. Li et al. (17) instead extended the dominance-based NSGA-II paradigm itself, improving its population initialization, selection, and mutation operators alongside a greedy repair strategy for high-dimensional feature selection in a method termed E-NSGA-II. Departing from all of the algorithm-level refinements above, Feng et al. (18) reformulated high-dimensional multiobjective feature selection as an evolutionary multitasking problem, solving multiple auxiliary problem formulations in parallel with a knowledge-transfer mechanism between them. Validated on 26 datasets, it illustrates that search-strategy innovation for this problem is not confined to refining any one of the four paradigms we compare, but it can also come from restructuring the search itself.

The remainder of this study details the data and methods in the Materials and Methods section; reports the extraction quality screening, feature selection, and validation results in the Results section, and discusses their implications and limitations in the Discussion section before concluding.

## 2 Materials and methods

Figure 1 summarizes the full LEAF pipeline; the following subsections detail each stage.

**Figure 1:**
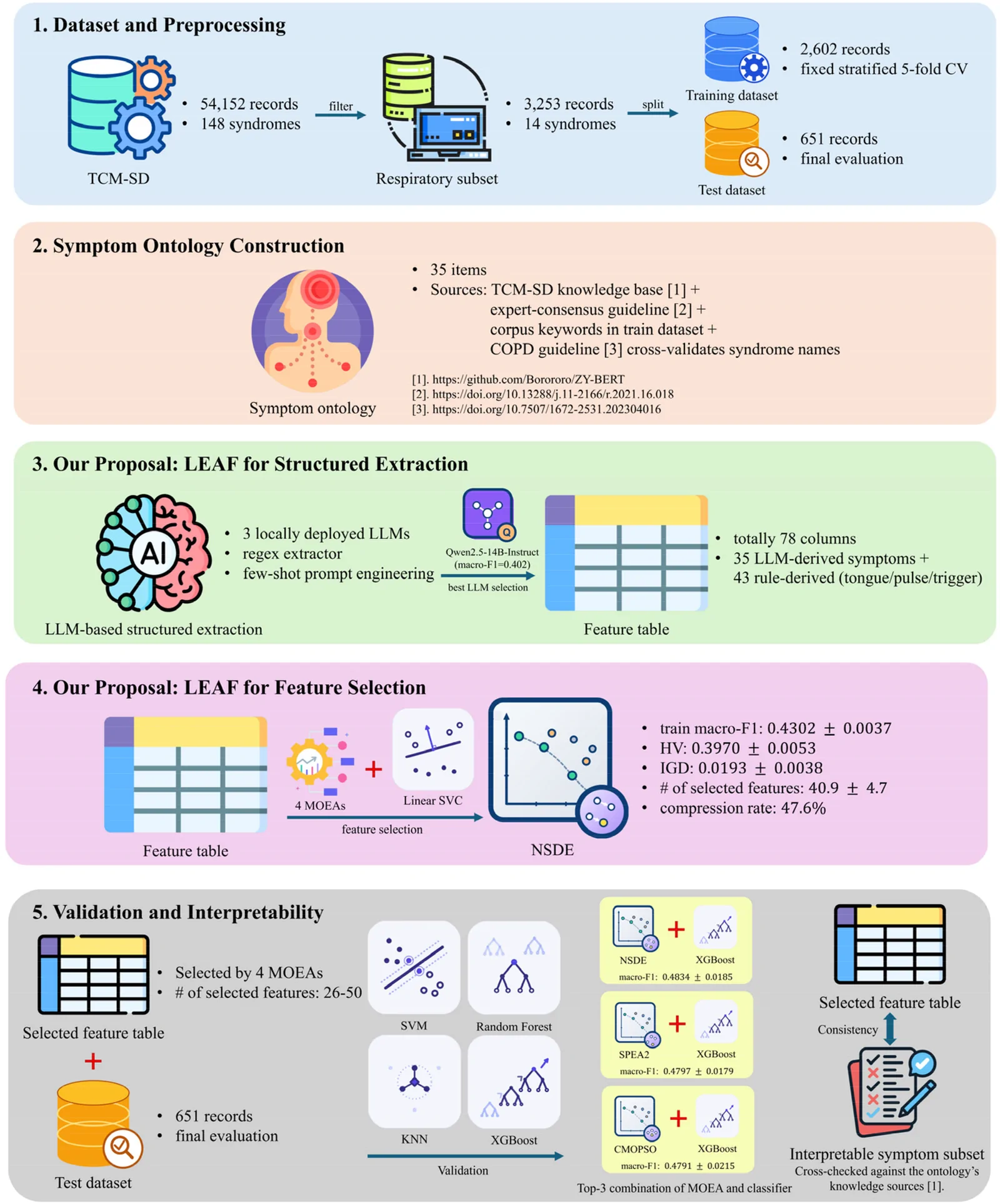
A LEAF pipeline overview: TCM-SD records are filtered to a respiratory subset and split into a training dataset under fixed fivefold cross-validation (CV) and a held-out test dataset, as detailed in Sections 2.1–2.2. A 35-item symptom ontology was built from the TCM-SD knowledge base, two expert-consensus guidelines, and corpus keywords, as detailed in Section 2.3. Three LLMs and a rule-based extractor produced per-pipeline feature tables, which were screened by a downstream proxy evaluation described in Sections 2.4–2.5. Four MOEAs selected feature subsets into Pareto fronts according to Section 2.7. Representative solutions are validated on four classifiers and compared with Friedman/Nemenyi tests according to Section 2.8. The resulting features are checked against the ontology's knowledge sources for interpretability according to Section 3.5. Icons from Flaticon: “TCM-SD” by bsd, “Respiratory subset”, “Symptom ontology”, “LLM-based structured extraction”, “Feature table”, “Selected feature table” by Magnific, “4 MOEAs” by SBTS2018, “Interpretable symptom subset” by Vectors Tank, licensed under Flaticon License.

### 2.1 Data source, respiratory disease subset, and ethics statement

TCM-SD (1) comprises 54,152 real-world clinical records covering 148 normalized syndromes, which were released under the MIT license for the code and CC BY-NC-SA 4.0 for the data available at https://github.com/Borororo/ZY-BERT. Each record carries a chief complaint, a free-text history of present illness, a four-diagnosis examination field, a Chinese disease name, and a normalized syndrome label. We filtered records by disease-name keyword matching against nine respiratory categories: cough, wheezing dyspnea, dyspnea, lung distension, lung atrophy, lung abscess, common cold, and wind-warmth lung-heat disease and infantile pneumonia–cough–wheeze disease, with the latter two being TCM equivalents of pneumonia. In the original Chinese records, these correspond to kesou, xiaobing, chuanzheng, feizhang, feiwei, feiyong, and ganmao, respectively; lung abscess (feiyong) returned zero matching records and is therefore absent from the subset. This yielded 3,255 records (6.01% of TCM-SD), which decreased to 3,253 after two duplicate encounters were removed. The subset spans 30 syndromes with a heavily skewed distribution: the two largest syndromes—phlegm dampness obstructing the lung and wind cold invading the lung—account for 80.5% of the records; the 17 syndromes with fewer than 10 samples were merged into a single “other/rare respiratory syndrome” class, yielding 14 modeling classes. Table 1 reports the resulting per-class record counts and proportions.

**Table 1:** Syndrome-class distribution of the respiratory-disease subset (3,253 records and 14 modeling classes after merging 17 syndromes with fewer than 10 samples into “other/rare respiratory syndrome”).

Syndrome	Records	Share (%)
Phlegm dampness obstructing the lung	2,058	63.26
Wind cold invading the lung	562	17.28
Phlegm heat obstructing the lung	138	4.24
Phlegm stasis interlocking	108	3.32
Wind heat invading the lung	74	2.27
Lung–kidney qi deficiency	71	2.18
Other/rare respiratory syndrome	65	2.00
Lung-defense (wei) qi insecurity	48	1.48
Qi deficiency with blood stasis	42	1.29
Yang deficiency with water flooding	24	0.74
Phlegm-dampness internal obstruction	22	0.68
Kidney failing to grasp qi	16	0.49
Cold phlegm obstructing the lung	13	0.40
Qi and blood deficiency	12	0.37
Total	3,253	100

The original syndrome labels of TCM-SD are recorded in Chinese; the English names in this table are translations provided by us for readability.

This study reused the publicly available, previously deidentified TCM-SD dataset (1); no new human subject data were collected, and the ethics statement of the original dataset reported that all records were desensitized prior to release. Therefore, no additional ethical approval or informed consent was required for this study.

### 2.2 Data split

We drew a stratified 80/20 split (random seed 42), stratified on the 14-class label into a 2,602-record training dataset and a 651-record held-out test dataset, with the latter touched only once, at final classifier validation. Within the training dataset, we fixed a stratified five-fold assignment that is reused, unchanged, by every LLM pipeline and every wrapper fitness evaluation of MOEA so that all comparisons in this paper share the same folds.

### 2.3 Symptom ontology

The ontology was built from three sources. First, the syndrome knowledge base of TCM-SD contributed 232 candidate phrases drawn from the *Typical_performance* field of the 30 respiratory syndromes. Second, the 2021 Expert Consensus on TCM Diagnosis and Treatment of Cough (19) contributed four symptom terms, which were added directly: nasal flaring, which the TCM-SD knowledge base also attests, and three terms absent from it; mouth-open shoulder-raising; hoarse wheeze sound; and rapid onset and resolution. Third, unsupervised keyword extraction was performed over the free text of the training dataset using the jieba segmenter. Every one of the 35 resulting symptom terms is traceable to at least one of these three sources. Supplementary Material S1 lists each term alongside their sources it is traceable to.

The 2022 Integrated Chinese and Western Medicine Guideline for chronic obstructive pulmonary disease (COPD) (20) played a complementary, syndrome-level role rather than contributing individual symptom terms: we crosschecked its documented syndrome names and diagnostic criteria against the syndrome labels of TCM-SD, confirming, for example, that wind cold invading the lung and phlegm heat obstructing the lung are named consistently across both sources. This served as an independent sanity check on the syndrome taxonomy of the respiratory subset rather than as a source of ontology terms.

This process also surfaced a data-quality finding relevant to prompt design: the four-diagnosis field is approximately 80% fixed physical-examination boilerplate, with diagnostically useful content concentrated in a tongue/pulse tail. Therefore, we route tongue, pulse, and disease-trigger information (e.g., cold exposure) through regex rules rather than through the LLM and feed the LLM only the chief complaint and the history-of-present-illness text. The resulting ontology comprises 35 symptom items for LLM extraction in addition to rule-extracted tongue color, coating color, coating quality, pulse, and trigger features.

### 2.4 LLM-based structured extraction

For each record, an LLM is given the chief complaint, history text, the full 35-item symptom list with synonyms, and four few-shot examples, which were drawn from real training dataset cases with their TCM-SD syndrome labels but were annotated by the project team against the ontology and guideline sources rather than by a clinician. Then, they were asked to return, for every symptom, one of {present, absent, not mentioned} plus a severity descriptor as schema-constrained JSON (JavaScript Object Notation) via guided JSON decoding of vLLM (21), which is built on finite-state, machine-guided constrained decoding (22). Three LLMs were used for extraction: two general-purpose models—Qwen2.5-14B-Instruct (6) and Phi-4 (23)—and one medical/TCM domain model—BianCang–Qwen2.5-7B-Instruct (8). On a 21-case–stratified sample, Qwen2.5-14B-Instruct and Phi-4 reached 100% JSON-format compliance, with the latter achieving compliance only after stripping a markdown code fence that some completions added despite constrained decoding, and BianCang reached 90% JSON-format compliance. All three models were then run on the full training dataset and held-out test dataset, reaching 96.0%–100% valid JSON coverage. The full prompt, including the system instructions, verbatim Chinese text, all four few-shot examples, and the JSON output schema, is reproduced in Supplementary Material S2.

### 2.5 Extraction-quality screening

We substituted a downstream proxy evaluation: the feature table of each LLM pipeline is scored by macro-F1 from a fixed-fold, class-balanced linear support vector machine (SVM) using the same fold assignment as mentioned in Section 2.2, and pipelines were ranked by this score rather than by symptom-level agreement with a human annotator. Macro-F1 is the unweighted mean of the per-class F1 score, which is computed as follows:

$$\mathrm{macro}-F1=(1/C)\Sigma_{c=1}^{C}\;F1_{c},F1_{c}=2\;\text{·}{\mathrm{precision}}_{c}\;\text{·}{\mathrm{recall}}_{c}/({\mathrm{precision}}_{c}+{\mathrm{recall}}_{c})$$

where *C* = 14 is the number of syndrome classes described in Section 2.1 and precisionc and recallc treat class c as the positive class. Averaging the 14 per-class scores unweighted, rather than weighting by class size, is why macro-F1 is a metric considered suitable for class-imbalanced settings (24). We report the *post-hoc* consistency check for this substitution in Section 3.3.

### 2.6 Feature table construction

The symptom judgments of three LLM pipelines—present mapped to 1 and absent or not-mentioned mapped to 0—are combined with the rule-extracted tongue/pulse/trigger features into one 78-column table per pipeline: 35 LLM-derived symptom columns and 43 rule-derived columns. Table 2 presents a real six-row excerpt from the training dataset table of the Qwen2.5-14B-Instruct pipeline (2,596 of the 2,602 training-dataset records, with the shortfall being records for which the LLM of this pipeline did not return valid JSON output, according to Section 2.4), spanning three syndrome classes and a representative subset of columns; at up to 2,602 rows by 78 feature columns per pipeline-split combination, the full tables are too large to typeset and are instead provided as the six CSV files described in Supplementary Material S3, with one per LLM pipeline (Qwen2.5-14B-Instruct, Phi-4, and BianCang–Qwen2.5-7B-Instruct) and data split (training dataset and held-out test dataset).

**Table 2:** Excerpt from the training-dataset feature table of the Qwen2.5-14B-Instruct pipeline: six real rows and a representative subset of columns (the full table with 2,596 rows and 78 columns is available in Supplementary Material S3).

Case ID	Syndrome	Fold	Cough	Expect.	Fever	Panting	Tongue red	Pulse slip.	Trigger: cold
479991	Qi deficiency with blood stasis	4	0	0	0	1	1	1	0
464235	Phlegm dampness obstructing the lung	1	1	1	0	1	1	0	1
443955	Phlegm dampness obstructing the lung	1	1	1	0	1	1	1	1
457228	Phlegm dampness obstructing the lung	0	1	1	0	1	1	1	1
449548	Phlegm stasis interlocking	1	1	1	0	0	1	1	1
429413	Phlegm dampness obstructing the lung	3	1	1	0	1	0	1	1

### 2.7 Multiobjective evolutionary feature selection

We compare four MOEAs spanning distinct search paradigms: dominance-based NSGA-II (11), archive-based SPEA2 (25), competitive swarm–based CMOPSO (26), and differential evolution–based NSDE, which pairs differential evolution's mutation and crossover operators (12) with non–dominated sorting survival selection of NSGA-II (11), implemented via pymoo 0.6.2 (27). Beyond the population size of 100 shared by all four algorithms, each retains default internal parameters of pymoo, which are summarized in Table 3, because no algorithm-specific tuning was performed. Defaults were retained deliberately: they give every algorithm the same untuned starting point, whereas tuning each algorithm separately would make the comparison depend on how much tuning effort each one received.

**Table 3:** Distinctive internal parameters of the four MOEAs, all left at their pymoo 0.6.2 defaults; population size (100) is reported in the main text rather than being repeated here.

Algorithm	Distinctive internal parameters
NSGA-II	Simulated binary crossover (*η* = 15, *p* = 0.9), polynomial mutation (*η* = 20, *p* = 0.9), binary tournament selection, survival by non-dominated sorting, and crowding distance
SPEA2	Simulated binary crossover (*η* = 15, *p* = 0.9), polynomial mutation (*η* = 20, *p* = 0.9), and survival by strength/density fitness with a normalized, size-truncated archive
CMOPSO	A maximum velocity rate of 0.2, an elite archive size of 10, random initial particle velocities, a polynomial mutation perturbation rate of 0.5, and survival by crowding distance tournament among elites
NSDE	Differential evolution variant DE/rand/1/bin, crossover rate (CR) = 0.7, scale factor F randomized in (0,1) with jitter *γ* = 10^–4^, bounce-back boundary repair, survival by non-dominated sorting, and crowding distance

Because CMOPSO and NSDE are continuous-domain algorithms, all four algorithms optimized the same representation for a fair comparison: a continuous vector x in [0,1]^78^, thresholded at 0.5 to obtain the binary feature mask evaluated by the fitness function, which is a standard representation for applying continuous metaheuristics to feature selection (10). Two objectives were optimized: macro-F1, which was maximized by minimizing its negative since pymoo optimizes by minimization, and the selected-feature ratio, which was minimized directly; both were computed via the fixed five-fold linear SVM evaluator described in Section 2.5. A third, optional knowledge base–consistency objective was not tractable to be defined as a single global optimization target shared across 14 classes and is instead reported *post hoc* on the final selected subsets described in Section 3.5. Each algorithm was run with a population size of 100 for 100 generations, resulting in 10,000 fitness evaluations, and was repeated across 10 random seeds (2,026 + 91*i* for *i* = 0,…,9) with the same population size, evaluation budget, and number of independent runs as those used for the NSGA-II baseline in a comparable multiobjective feature selection study (28). For each algorithm, the seed whose best macro-F1 Pareto solution attains the median macro-F1 across the 10 seeds, taken as the upper of the two middle-ranked seeds as 10 is even, is taken as that algorithm's representative solution: a single, concrete feature subset used for the classifier panel comparison, the cross-LLM-pipeline check in Section 3.3, and the knowledge-consistency check in Section 3.5, avoiding a best-of-10 selection bias when one inspectable feature list is required.

We compared the four algorithms with three seed-blocked Friedman’s tests, 10 seeds as blocks and four algorithms as treatments, which is the standard design for comparing algorithms across repeated runs (29): one on the best-solution training-fold macro-F1 of each algorithm; one on hypervolume (30) with reference point (0, 1.05) in the (−macro-F1, feature ratio) objective space, which scores the convergence and diversity of the whole Pareto front instead of a single point, corroborated by an analogous test on the inverted generational distance (IGD) (31); and one on held-out macro-F1, obtained by retraining and evaluating each of the best macro-F1 solutions of 10 seeds once on the held-out test dataset and by averaging over the four downstream classifiers per seed so that held-out generalization is similarly summarized by a 10-seed mean rather than by a single point estimate. Formally, for a biobjective front *S* and a reference point r dominated by every point in *S*, hypervolume is the Lebesgue measure of the region jointly dominated by *S* and bounded by r:

$$\mathrm{HV}(S,r)=\Lambda (\cup \{s\in S\}\{z\in R^{2}:s\underline{≺}z\underline{≺}r\})$$

where Λ(·) denotes the Lebesgue measure and *s* ⪯ *z* denotes component-wise dominance; instead, IGD measures how well a front covers a reference set *P*—the non-dominated union of fronts of all 40 runs reported in Section 3.2—as the mean distance from each reference point to its nearest approximating point:

$$\mathrm{IGD}(S,P)=(1/|P|)\Sigma (p\in P)\min(s\in S)∥p-s{∥}_{2}$$

Training-fold and held-out macro-F1 are outcome-relevant metrics as they measure the classification performance based on which the pipeline is ultimately judged, whereas hypervolume and IGD summarize the search process itself. Therefore, we prioritized the two outcome-relevant metrics over hypervolume and IGD when they disagree, treating the latter as a secondary diagnostic of search quality rather than as the deciding criterion. NSDE attains the best mean on both outcome-relevant metrics and is accordingly treated as the primary algorithm for the remaining comparisons. Selected-feature stability is reported as the fraction of the best F1 solutions of the 10 seeds that include each feature, averaged over all features ever selected by this algorithm.

### 2.8 Downstream validation and statistical comparison

From the median-seed representative solution of each algorithm, we retrained on the full training dataset and evaluated once on the held-out test dataset with four classifiers: a radial basis function (RBF)-kernel SVM (32), random forest (33), K-nearest neighbors (KNN), and XGBoost (34), which were all implemented via scikit-learn (35) except XGBoost. It is worth noting that this RBF-kernel SVM is distinct from the linear-kernel LinearSVC used before in this study as the search-time fitness evaluator.

The training time and validation classifiers deliberately differed. The search-time linear SVM is a fast surrogate that was needed because each MOEA run evaluates thousands of candidate feature masks, whereas, here, the representative solution of each algorithm was evaluated only once. This affordability allows a broader panel of classifiers with different inductive biases (kernel-based, tree-ensemble, and distance-based), ensuring that the apparent benefit of a selected subset is not merely an artifact of the one classifier against which it was optimized, a design mirrored by other evolutionary feature selection studies that validate selected subsets against classifiers distinct from the search-time fitness function (36, 37).

We compared methods—the four MOEAs and full-feature and random-subset baselines—using a Friedman test across the four classifiers as blocks, followed by Nemenyi *post-hoc* pairwise comparisons (29). This single representative solution comparison is distinct from, and complements, the 10 seed–averaged held-out comparison that was used to identify NSDE as the primary algorithm reported in Section 2.7.

To examine class imbalance, we also reported the F1 score of each of the 14 classes, both on the held-out test dataset and from out-of-fold predictions over the fixed five training folds of Section 2.2, together with a minority-class macro-F1, the unweighted mean F1 over the 12 classes other than the two majority syndromes. The search-time LinearSVC and the validation SVM and random forest already use class-balanced weights. We compared this original configuration with two imbalance-handling variants that were applied only when training the four validation classifiers: a cost-sensitive variant that additionally gives XGBoost class-balanced sample weights, leaving KNN unchanged because it has no class-weight option, and random oversampling of every training class to the size of the largest class (random seed 42), before fitting all four classifiers. We did not use the synthetic minority oversampling technique (SMOTE) whose linear interpolation between neighboring records would create non-binary values for the binary features used here. These analyses reused 10 best macro-F1 solutions of each algorithm from Section 2.7 and did not repeat the evolutionary search.

## 3 Results

### 3.1 Extraction pipeline screening

On the training dataset, the fixed-fold proxy evaluation ranked Qwen2.5-14B-Instruct the highest at a macro-F1 of 0.4015 ± 0.0275, slightly ahead of Phi-4 at a macro-F1 of 0.3990 ± 0.0359, with the TCM domain model BianCang–Qwen2.5-7B-Instruct clearly being the lowest at a macro-F1 of 0.3579 ± 0.0307. Therefore, Qwen2.5-14B-Instruct was fixed as the extraction pipeline for the feature-selection comparison in Section 3.2. Figure 2 shows the five-fold-level scores behind each mean: ranges of Qwen2.5-14B-Instruct and Phi-4 overlap substantially, consistent with their close means, while five folds of BianCang sit systematically lower on average despite its single best fold still overlapping with the weakest folds of two general-purpose models.

**Figure 2:**
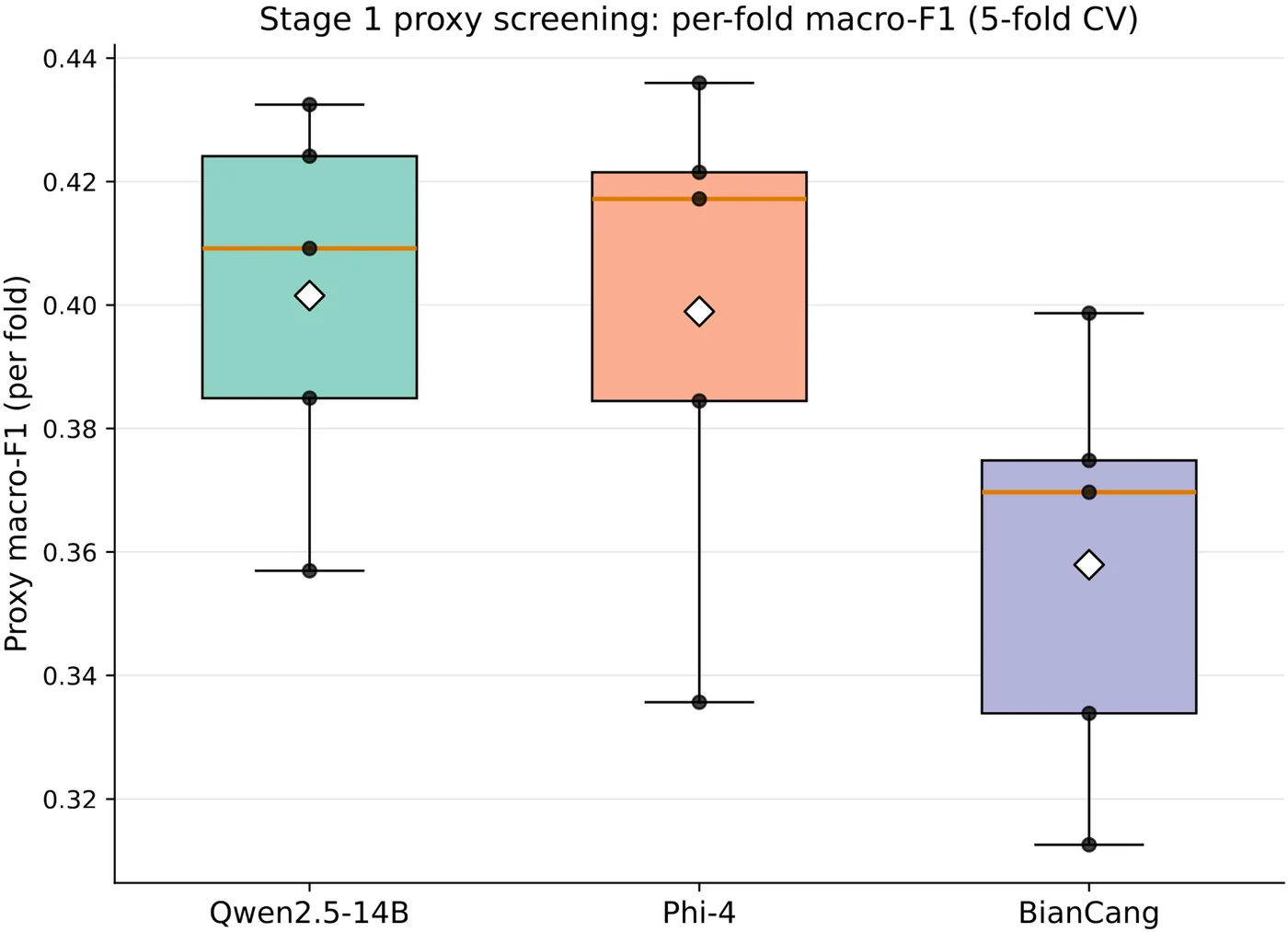
Proxy macro-F1 for each of the five crossvalidation folds with one pipeline per box; diamonds mark the mean.

### 3.2 Multiobjective feature selection

Table 4 reports, for each of the four MOEAs, the training-fold macro-F1, feature count, and held-out macro-F1 across 10 random seeds, which are expressed as mean ± SD. The full-feature and random-subset controls are reported alongside them, with each evaluated only once: the former because they are deterministic and the latter because they reflect a single fixed draw. Across the 10 seeds, the training-fold fitness values of the four MOEAs were tightly clustered, spanning 0.4255–0.4302, and each algorithm reduced the feature count by an average of 48%–60% relative to the full set of 78 features.

**Table 4:** MOEAs, with mean ± SD feature count, training-fold macro-F1, and held-out macro-F1 across 10 seeds, vs. full-feature and random-subset controls, sorted by held-out macro-F1.

Method	Features	Reduction (%)	Train-fold macro-F1	Held-out macro-F1
Full features	78	0	-	0.4663
NSDE	40.9 ± 4.7	47.6	0.4302 ± 0.0037	0.4334 ± 0.0120
CMOPSO	38.2 ± 3.3	51.0	0.4255 ± 0.0037	0.4293 ± 0.0175
SPEA2	31.6 ± 3.1	59.5	0.4293 ± 0.0070	0.4258 ± 0.0088
NSGA-II	34.5 ± 3.7	55.8	0.4297 ± 0.0060	0.4234 ± 0.0087
Random subset	26	66.7	-	0.3188

Figure 3 plots the median-seed Pareto front of each algorithm in the feature-ratio/training-fold-macro-F1 plane. NSGA-II and SPEA2 trace visibly smoother, more continuous fronts across the full feature-ratio range; the CMOPSO front is sparser and sits below the others through the low-to-mid feature–ratio range, consistent with its markedly worse hypervolume and IGD reported below; and the NSDE front is comparable with those of NSGA-II and SPEA2 through most of the range and extends further than both into high feature ratios.

**Figure 3:**
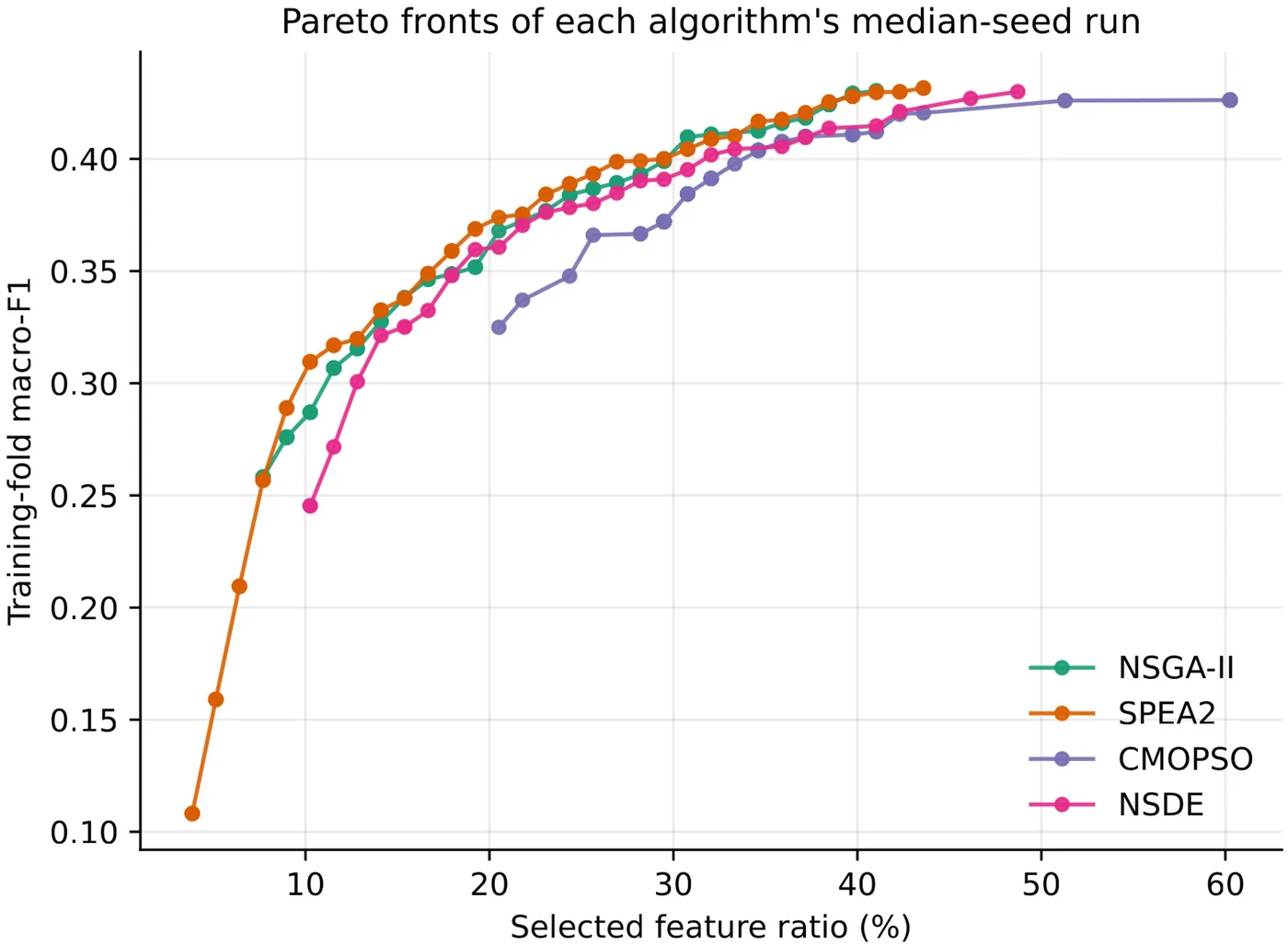
Pareto fronts of the median-performing seed of each algorithm, by training-fold macro-F1, in the feature-ratio/macro-F1 plane.

As described in Section 2.7, we compare the 10-seed results of the four algorithms using three seed-blocked Friedman’s tests, prioritizing the two outcome-relevant metrics over the two search process metrics whenever they disagree. On best-solution training-fold macro-F1, the four algorithms did not differ significantly (*χ*^2^ = 2.52, *p* = 0.47), with NSDE nominally being the best. On held-out macro-F1, averaged similarly over the 10 seeds, the four algorithms again did not differ significantly (*χ*^2^ = 3.00, *p* = 0.39), with NSDE again nominally being the best at 0.4334 ± 0.0120, ahead of CMOPSO (0.4293 ± 0.0175), SPEA2 (0.4258 ± 0.0088), and NSGA-II (0.4234 ± 0.0087). Since NSDE attains the best mean on both outcome metrics, its median-seed representative solution (at 38 features) is carried forward to the classifier-panel comparison below.

With regard to hypervolume, with reference point (0, 1.05), which scores the entire Pareto front of each algorithm rather than a single best point, the difference was significant (*χ*^2^ = 20.28, *p* = 0.0001). NSGA-II attained the best mean hypervolume (0.4025 ± 0.0038), ahead of NSDE (0.3970 ± 0.0053) and SPEA2 (0.3967 ± 0.0061); Nemenyi comparisons found CMOPSO (0.3518 ± 0.0105) to be significantly worse than each of the other three algorithms (*p* ≤ 0.017), with no significant difference among NSGA-II, SPEA2, and NSDE. The IGD measured against the non-dominated union of all fronts of 40 runs was in agreement with the hypervolume result: NSGA-II had the lowest mean IGD (0.0125 ± 0.0030), followed by SPEA2 (0.0171 ± 0.0063) and NSDE (0.0193 ± 0.0038), with CMOPSO clearly being the worst (0.0644 ± 0.0131). We return to this disagreement between search process and outcome metrics in the Discussion section.

Figure 4 shows the full 10-seed distribution behind each of these four means: the CMOPSO spread on hypervolume and IGD is visibly wider and worse-centered than that of the other three algorithms, while its held-out macro-F1 distribution, although also being wide, is centered no lower than that of NSGA-II or SPEA2, concretely illustrating that a poorly explored front does not translate into poor held-out classification.

**Figure 4:**
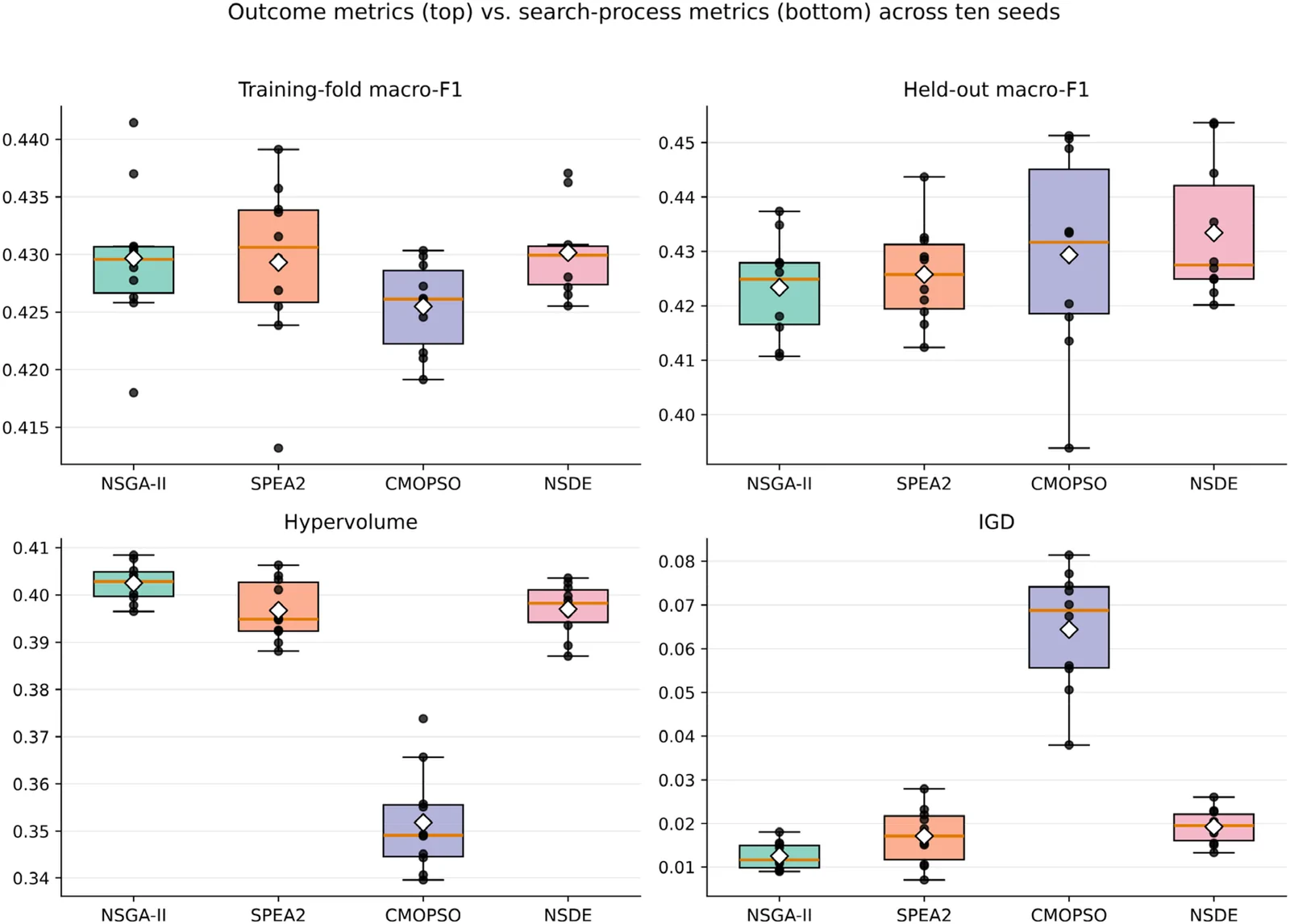
Distribution across 10 seeds of training-fold macro-F1, held-out macro-F1, hypervolume, and IGD for each algorithm; diamonds mark the mean, and horizontal lines indicate the median.

Figure 5 breaks the held-out column in Table 4 down by classifier: XGBoost and random forest are the strongest classifiers for every algorithm, KNN is the weakest by a wide margin, and the advantage of NSDE over the other three algorithms is driven mainly by its SVM and random forest scores rather than by a uniform lift across all four classifiers; its XGBoost lead is marginal, and its KNN score is the lowest of the four algorithms.

**Figure 5:**
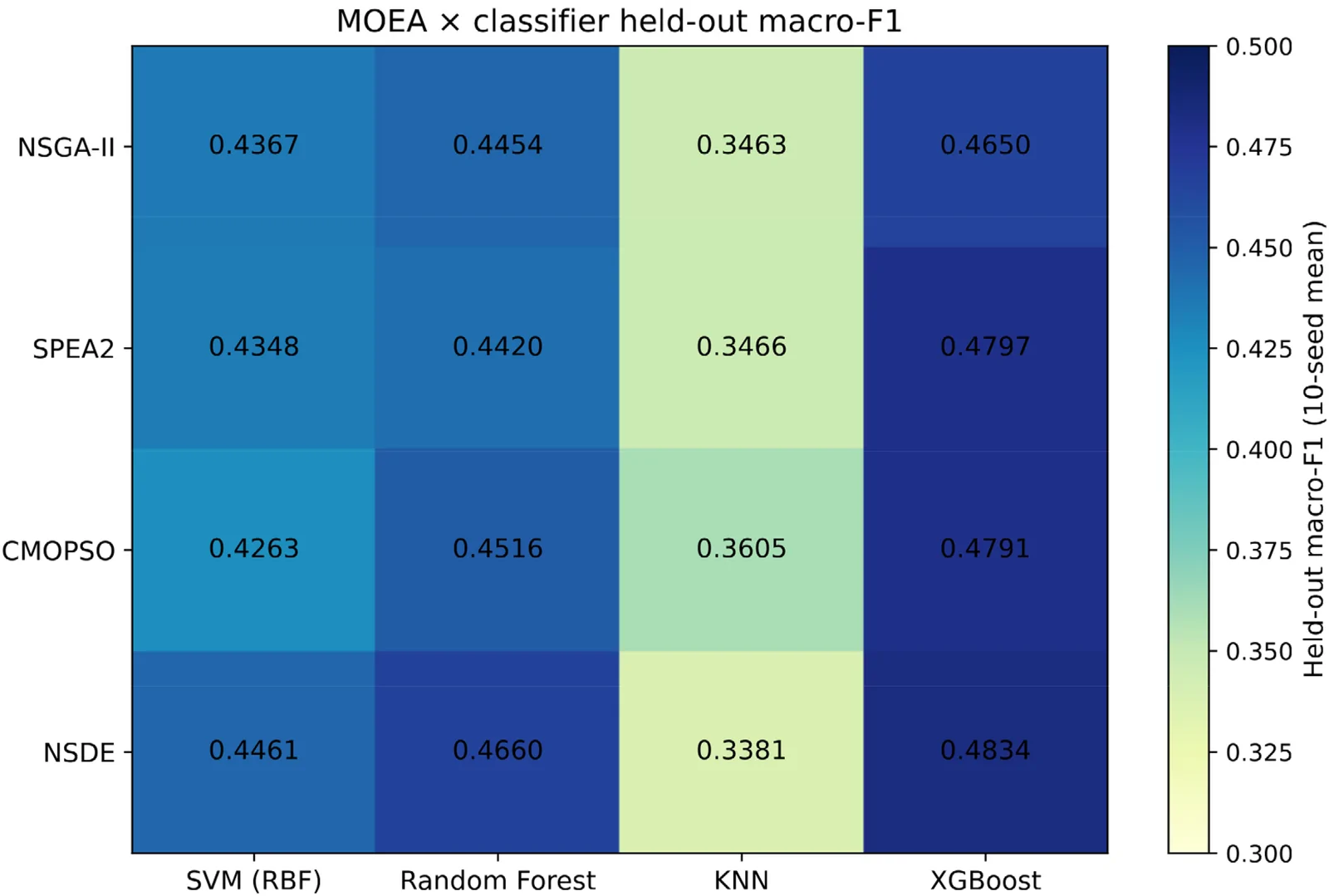
Held-out macro-F1, averaged over 10 seeds, for each combination of evolutionary algorithm and downstream classifier.

A Friedman test across the four downstream classifiers, comparing the median-seed representative solutions of all four algorithms together with the full-feature and random-subset controls, found a significant overall difference among the six methods (*χ*^2^ = 13.86, *p* = 0.017). However, Nemenyi’s *post hoc* comparisons found no significant difference between any evolutionary algorithm and the full-feature baseline (*p* = 0.298–0.975).

Among comparisons against the random-subset control, no representative solution of any evolutionary algorithm was significantly better (CMOPSO, *p* = 0.087; NSDE, *p* = 0.207; SPEA2, *p* = 0.772; and NSGA-II, *p* = 0.531). The only significant pairwise difference was the full-feature baseline over the random-subset control (*p* = 0.009). On point estimates, the held-out macro-F1 of 0.4663 of the full-feature baseline in Table 4 exceeds the 10-seed mean of every algorithm by 0.033 for NSDE to 0.043 for NSGA-II. However, the classifier panel is too small (only four classifiers) to statistically confirm any ordering among the evolutionary algorithms or between them and the full-feature baseline; the random-subset control comprises a fixed random draw of one-third of the 78 features (26 features).

Selected-feature stability, the fraction of the best F1 solutions of the 10 seeds sharing each selected feature, averaged over all features ever selected, was the highest for SPEA2 (0.645) and lowest for CMOPSO (0.509), with NSGA-II (0.595) and NSDE (0.538) falling in this range.

### 3.3 Crossvalidating the proxy screening: LLM pipeline comparison

Repeating feature selection of NSDE on the feature tables of the other two LLM pipelines, using a single NSDE run per pipeline at the seed of the median-seed representative solution of NSDE on the Qwen2.5-14B-Instruct pipeline (2,663) and evaluating its best macro-F1 solution, reproduced the same ranking found in the extraction-quality screening described in Section 3.1: Qwen2.5-14B-Instruct led at 0.4258 with 38 features, ahead of Phi-4 at 0.4128 with 33 features and ahead of BianCang–Qwen 2.5-7B-Instruct at 0.3685 with 35 features, which were all evaluated once on the held-out test dataset, restricted to each pipeline's records with valid extractions (650, 651, and 625 of 651, respectively). The fact that this ranking, established without any human-annotated symptom-level gold standard, is reproduced after the full feature selection pipeline supports the use of proxy evaluation as a substitute for gold-standard annotation in this setting.

Figure 6 places the two stages side by side: the ordering is unchanged. The stage 2 score is higher than the 78-feature proxy score for every pipeline, but the two stages differ in classifiers and evaluation data, so this difference should not be read as a gain from feature selection.

**Figure 6:**
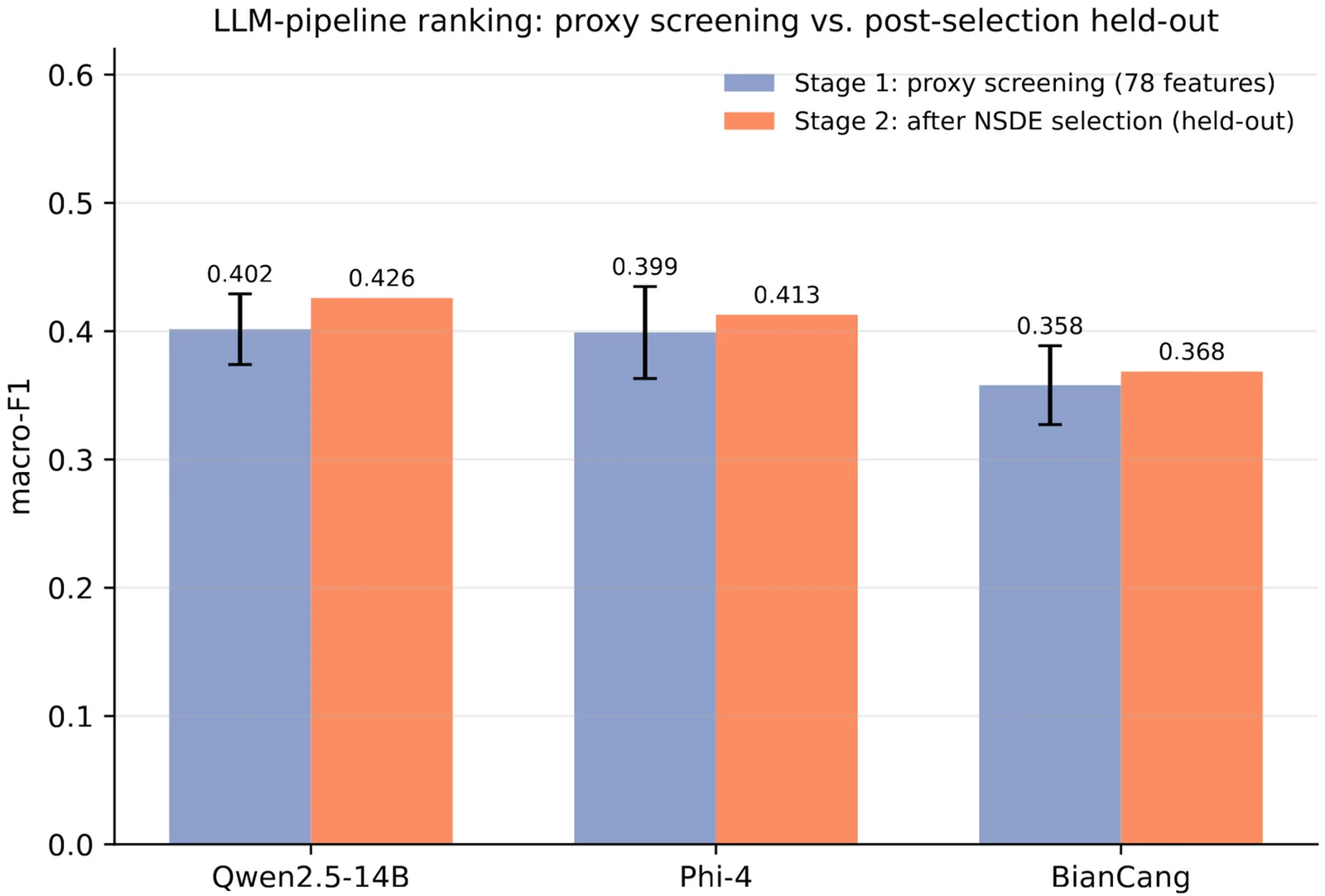
LLM pipeline macro-F1 at stage 1: the fixed-fold proxy screen on all 78 features, with error bars showing the five-fold SD. LLM pipeline macro-F1 at stage 2: held-out macro-F1 after NSDE feature selection, averaged over four classifiers.

Figure 7 breaks the stage 2 score of each pipeline down by classifier: the same XGBoost-best, KNN-worst pattern observed for the four MOEAs in Figure 5 holds within every LLM pipeline, and the representative solution of Qwen2.5-14B-Instruct is ahead of the other two pipelines on SVM and random forest. In contrast, the pattern of Phi-4 is ahead on KNN and XGBoost, and therefore, Qwen2.5-14B-Instruct, on average, leads but not on every classifier.

**Figure 7:**
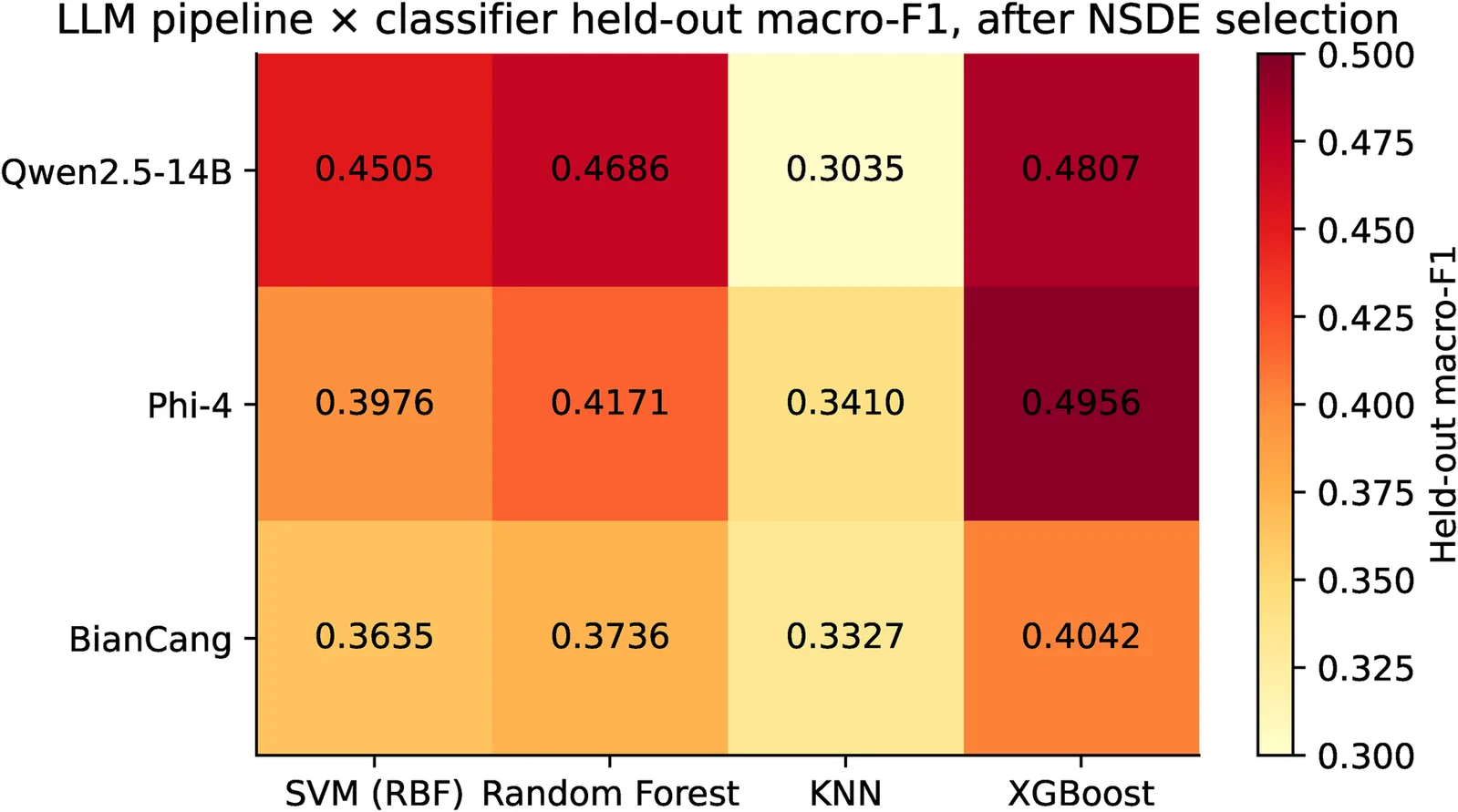
Held-out macro-F1 for the NSDE-selected feature subset of each LLM pipeline by downstream classifier.

### 3.4 NSDE feature selection stability

Across the 10 seeds, the best macro-F1 Pareto solution of NSDE selected 76 of the 78 candidate features at least once, but with markedly uneven reliability. Table 5 lists the 20 features selected in every one of the 10 seeds; together with eight further features selected in eight or nine of the 10 seeds, these 28 features, 36% of the 78 candidates, met a ≥80% per-feature selection-frequency threshold. This per-feature view is distinct from, and more granular than, single algorithm–level selected-feature stability figure of NSDE of 0.538 already reported in Section 3.2, which averages selection frequency across all 76 ever-selected features rather than counting how many cleared the 80% bar. At the other end, 41 of the 76 ever-selected features appeared in half of or fewer than the 10 runs, indicating that the 38-feature representative solution of NSDE combines a consistently reselected core with a substantial share of seed-dependent, lower-confidence features. The full selection count for all 76 ever-selected features is given in Supplementary Material S4.

**Table 5:** The 20 features selected by the best macro-F1 solution of NSDE in all 10 seeds.

Feature	Category
Orthopnea	Symptom
Abnormal urination/defecation	Symptom
Difficulty breathing	Symptom
Panting/dyspnea	Symptom
Edema	Symptom
Sputum color	Symptom
Sputum consistency	Symptom
Fatigue	Symptom
Reduced appetite	Symptom
Tongue color: purple	Rule (tongue)
Coating color: white	Rule (coating)
Coating color: yellow	Rule (coating)
Coating quality: thin	Rule (coating)
Pulse: deep	Rule (pulse)
Pulse: rapid	Rule (pulse)
Pulse: wiry	Rule (pulse)
Pulse: thready	Rule (pulse)
Pulse: weak	Rule (pulse)
Trigger: cold exposure	Rule (trigger)
Trigger: seasonal change	Rule (trigger)

The always-selected core is dominated by systemic and respiratory symptoms (orthopnea, dyspnea, edema, fatigue, and reduced appetite) alongside sputum quality descriptors and five distinct pulse qualities, a combination consistent with features that discriminate broadly across the 14 syndrome classes rather than with markers that are specific to a single rare syndrome. All nine of the LLM-derived symptom terms of the always-selected core are independently attested against the TCM-SD knowledge base by the substring check described in Section 3.5, including reduced appetite, whose ontology entry (Supplementary Material S1) traces to corpus keyword extraction rather than to the knowledge base directly. By contrast, the four symptom terms added directly from the cough expert-consensus guideline (Supplementary Material S1), three of which are absent from the TCM-SD knowledge base (nasal flaring, mouth-open shoulder-raising, hoarse wheeze sound, and rapid onset and resolution), all fall in the lower half of the stability distribution of NSDE, selected in 4, 3, 3, and 3 of 10 seeds, respectively. With only four such terms and no control for how syndrome-specific each one is, this pattern is not strong evidence that knowledge-base attestation predicts selection stability. However, it is consistent with the fact that the least corroborated terms of ontology were also its least reliably useful terms for classification, and we return to this observation in the Discussion section.

### 3.5 Knowledge base consistency

Of the full 35-item symptom ontology, 32 terms (91.4%) were independently attested as a substring match against the syndrome knowledge base of TCM-SD. The remaining three (mouth-open shoulder-raising, hoarse wheeze sound, and rapid onset and resolution) were added solely from the cough expert-consensus guideline and carried no TCM-SD attestation, and therefore, 91.4% was the ceiling of this metric rather than being a property specific to any selection method.

Against this ceiling, the median-seed representative solution of NSDE on the Qwen2.5-14B-Instruct pipeline selected 38 features in total (16 LLM-derived symptom features and 22 rule-derived tongue/pulse/trigger columns), and all 16 symptom features were attested, a 100% consistency rate by this coarse, substring-attestation metric. However, this single-solution figure is not representative of the typical behavior of NSDE: repeating the same check on each of the best macro-F1 solutions of the 10 seeds gives a mean consistency of 0.955 ± 0.039 (in the range of spanning 0.905–1.000), with the three guideline-only terms appearing in varying combinations across the selections of six of the 10 seeds; the median-seed representative solution happens to be one of the four seeds that avoided all three terms.

Because nearly every ontology term was itself derived from the same knowledge base or guideline sources that this check queries, and because even the ceiling rate falls below 100%, this check offers necessary but weak evidence of clinical plausibility rather than strong independent validation, a point that we returned to in the Discussion section. Figure 8 shows rates of all 10 seeds against the 91.4% ceiling: the median-seed representative solution, highlighted in pink, was one of the four seeds that reached 100%, whereas the other six ranged from 0.905 to 0.944, three of them below 91.4%.” As Figure 8 shows, three of the six seeds (0.938-0.944) lie above 91.4%.

**Figure 8:**
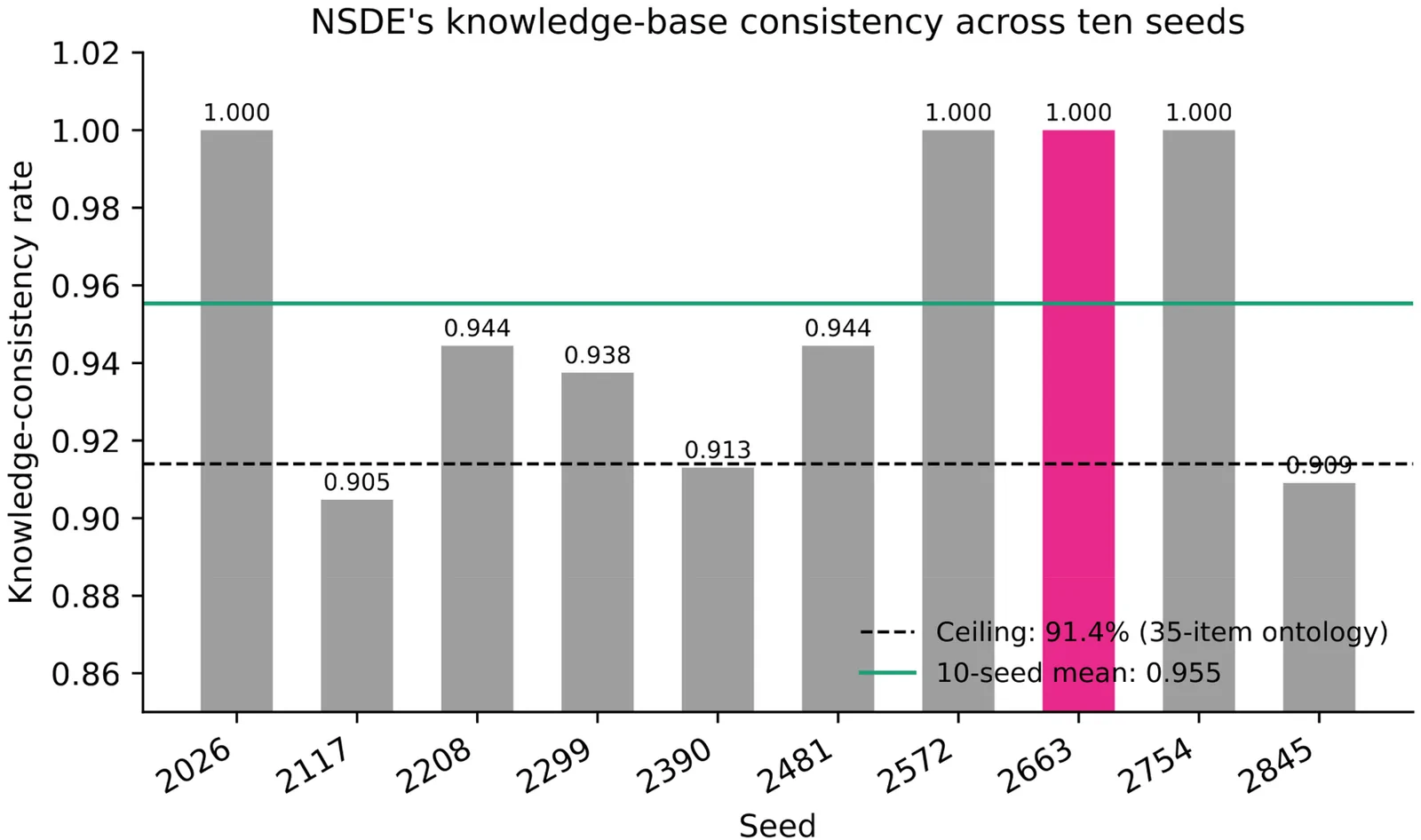
The NSDE knowledge-consistency rate for the best macro-F1 solutions of each of the 10 seeds against the 91.4% ceiling and the 10-seed mean; the bar highlighted in pink is the median-seed representative solution reported in the text.

### 3.6 Per-class performance and class-imbalance handling

Table 6 reports per-class F1 for NSDE, averaged over solutions of its 10 seeds and the four classifiers, and for the full-feature baseline. On the held-out test dataset, NSDE reached F1 of 0.805 and 0.620 on the two majority syndromes, whereas its F1 on the 12 minority classes ranged from 0.075 for phlegm dampness internal obstruction to 0.975 for qi and blood deficiency, the latter resting on only two held-out records. The held-out minority-class macro-F1 of NSDE was 0.386 vs. 0.425 for the full-feature baseline, and the other three algorithms were close to NSDE at 0.376–0.384. In the out-of-fold training estimates, which rest on four times as many minority-class records, the gap between NSDE and the full-feature baseline nearly vanished—at 0.381 vs. 0.383. Therefore, per-class values for classes with fewer than 10 held-out records were unstable and should be read together with the out-of-fold estimates.

**Table 6:** Per-class F1 of NSDE and the full-feature baseline on the held-out test dataset and in out-of-fold predictions over the five fixed training folds, which were averaged over the four classifiers and, for NSDE, over the best-macro-F1 solutions of its 10 seeds; *n* is the number of records of each class with a valid Qwen2.5-14B-Instruct extraction, and minority classes are the 12 classes other than the two majority syndromes.

Syndrome	Held-out *n*	Held-out F1, NSDE	Held-out F1, full features	Training *n*	Out-of-fold F1, NSDE	Out-of-fold F1, full features
Phlegm dampness obstructing the lung	412	0.805	0.819	1,646	0.790	0.807
Wind cold invading the lung	113	0.620	0.619	448	0.623	0.616
Phlegm heat obstructing the lung	27	0.312	0.352	111	0.313	0.329
Phlegm stasis interlocking	21	0.523	0.624	85	0.552	0.585
Wind heat invading the lung	15	0.336	0.302	59	0.343	0.326
Lung–kidney qi deficiency	14	0.282	0.308	57	0.299	0.298
Other/rare respiratory syndrome	13	0.190	0.240	48	0.157	0.113
Lung-defense (wei) qi insecurity	9	0.433	0.601	39	0.688	0.775
Qi deficiency with blood stasis	9	0.437	0.458	33	0.330	0.314
Yang deficiency with water flooding	5	0.221	0.217	19	0.275	0.314
Phlegm dampness internal obstruction	4	0.075	0.083	18	0.165	0.173
Kidney failing to grasp qi	3	0.706	0.750	13	0.309	0.252
Cold phlegm obstructing the lung	3	0.143	0.160	10	0.141	0.146
Qi and blood deficiency	2	0.975	1.000	10	0.997	0.974
Macro-F1, all 14 classes	650	0.433	0.467	2,596	0.427	0.430
Minority-class macro-F1, 12 classes	650	0.386	0.425	2,596	0.381	0.383

Table 7 compares the two imbalance-handling variants with the original configuration. Class-balanced weighting of XGBoost lowered held-out macro-F1 for every method, by 0.008–0.038, because it lowered macro-F1 of XGBoost from 0.477 to 0.429 averaged over all MOEA solutions. Random oversampling increased the held-out macro-F1 of NSDE from 0.433 to 0.447 and its minority-class macro-F1 from 0.386 to 0.409 and increased the full-feature baseline from 0.467 to 0.483 and from 0.425 to 0.443; for the other three algorithms, macro-F1 changed by −0.001 to 0.005 and minority-class macro-F1 increased by 0.007–0.013. The gain from oversampling came mainly from KNN, whose macro-F1 increased from 0.348 to 0.413 averaged over all MOEA solutions, whereas that of XGBoost fell from 0.477 to 0.435. Under all three configurations, the full-feature baseline stayed ahead of every algorithm and the random subset stayed well behind, and therefore, the comparison in Section 3.2 does not depend on how class imbalance is handled at the classifier stage. The original configuration values in Table 7 reproduce values in Table 4 precisely up to 0.001; the residual differences come from KNN whose choice among the many equidistant training records produced by binary features is not fixed across runs.

**Table 7:** Held-out macro-F1 and minority-class macro-F1 under the original classifier configuration and two class imbalance–handling variants: cost-sensitive, adding class-balanced sample weights to XGBoost, and random oversampling of the training data.

Method	Macro-F1, original	Macro-F1, cost-sensitive	Macro-F1, oversampling	Minority macro-F1, original	Minority macro-F1, cost-sensitive	Minority macro-F1, oversampling
Full features	0.4667	0.4591	0.4826	0.4246	0.4171	0.4432
NSDE	0.4328 ± 0.0123	0.4220 ± 0.0091	0.4470 ± 0.0118	0.3863 ± 0.0144	0.3769 ± 0.0105	0.4088 ± 0.0121
CMOPSO	0.4293 ± 0.0173	0.4158 ± 0.0169	0.4302 ± 0.0224	0.3841 ± 0.0202	0.3719 ± 0.0193	0.3908 ± 0.0238
SPEA2	0.4264 ± 0.0084	0.4123 ± 0.0112	0.4252 ± 0.0141	0.3805 ± 0.0091	0.3681 ± 0.0111	0.3880 ± 0.0135
NSGA-II	0.4233 ± 0.0095	0.4144 ± 0.0103	0.4282 ± 0.0211	0.3762 ± 0.0102	0.3692 ± 0.0106	0.3892 ± 0.0218
Random subset	0.3182	0.2803	0.3156	0.2812	0.2504	0.2923

MOEA values are mean ± SD over 10 seeds, with each averaged over the four classifiers; rows are ordered as in Table 4.

## 4 Discussion

The central empirical finding is that multiobjective evolutionary feature selection, applied to LLM-extracted symptom features, can remove, on average, 48%–60% of the candidate features without a statistically detectable loss relative to using all features, although, on point estimates, the ten-seed mean held-out macro-F1 of every algorithm in Table 4 falls below that of 0.4663 of the full-feature baseline and falls by 0.033 for NSDE to 0.043 for NSGA-II.

However, this point-estimate gap is not confirmed by the classifier-panel significance test, which compares the single median-seed representative solution of each algorithm across only four classifiers: no evolutionary algorithm differed significantly from the full-feature baseline nor from a random feature subset one-third the size of the full set, and the only significant pairwise difference was the full-feature baseline over that random control. This is consistent with the modest absolute macro-F1 values (with their range spanning 0.21–0.53 across individual classifier scores of all six methods), reflecting a genuinely hard 14-class problem with a highly skewed class distribution and, for several rare syndromes, only a handful of held-out examples.

A second finding worth emphasizing is the dissociation between the structural quality of a Pareto front and the classification outcomes it actually produced. We compared the four algorithms using three seed-blocked Friedman’s tests, each computed once per seed rather than through iterative requerying of the held-out set: on training-fold macro-F1, the difference was not significant (*p* = 0.47), with NSDE being nominally the best; similarly, on held-out macro-F1 (averaged over representative solutions of all 10 seeds rather than a single-point estimate), the difference was not significant (*p* = 0.39), with NSDE again being nominally best; and on hypervolume, the difference was significant (*p* = 0.0001), with NSGA-II being the best, a result corroborated by IGD.

The two outcome-relevant metrics, training-fold and held-out macro-F1, were in agreement with each other, and both favored NSDE. Because held-out macro-F1 is itself one of the two deciding metrics and the held-out score of NSDE was then reported as its headline result, this reuse carried a mild optimistic selection bias. The bias was distinct from classical test-set leakage because no parameters were fitted against the held-out labels, but it runs in the same direction; we discuss this further in this study as a limitation. By contrast, hypervolume and IGD favored NSGA-II, the algorithm with the worst mean held-out macro-F1 of the four at 0.4234 against that of NSDE at 0.4334, and reliably identified CMOPSO as the weakest searcher, even though the mean held-out macro-F1 of CMOPSO, at 0.4293, is the second best of the four.

Therefore, a more diverse, better-converged Pareto front is no guarantee of a better-generalizing classifier in this study: hypervolume and IGD summarize how thoroughly an algorithm explores the two-objective space it was given, not how well any resulting feature subset classifies unseen data, and here, the two diverged. This dissociation is not specific to our setting. Quality indicators such as hypervolume and IGD are, by construction, measures of convergence and diversity of a solution set relative to the objective space of search, not of any downstream property external to that space, and their disagreement with an actual criterion of interest of a decision-maker is a recognized limitation of indicator-based comparison in the wider multiobjective optimization literature (38, 39). It has also been documented empirically, both directly on the validation-vs.-test split of the search, where the Pareto-front membership and hypervolume contribution of a solution can invert between validation and test data (40), and in multiobjective biomarker selection, where the ranking of an algorithm by test-set hypervolume does not always match its ranking by classification accuracy once the choice of downstream classifier is varied (41). We read this as a substantive reason to prefer outcome metrics over search-process metrics when the two disagree, not as evidence that hypervolume and IGD are uninformative; markedly worse values of CMOPSO on both remain a meaningful signal about its narrower, less diverse front, a property that a larger population or more generations might improve without necessarily changing which algorithm ranks best on the outcome metrics.

One might expect this same dissociation to appear between training-fold macro-F1 and held-out macro-F1 themselves because the former is computed with a single linear SVM as the search-time fitness evaluator—with a fast surrogate needed because the search evaluates thousands of candidate feature masks—while the latter is averaged over four classifiers with different inductive biases deliberately so that the benefit of a selected subset is not merely an artifact of the one classifier it was optimized against.

However, in this study, these two agree: both of them nominally favor NSDE, and both fail to reach significance across the four algorithms, suggesting that whatever mismatch exists between the linear search surrogate and the more diverse validation panel was not large enough here to reorder the algorithms.

There remains a real methodological gap worth noting: a feature subset shaped around a linear decision boundary need not transfer equally well to a distance-based classifier such as KNN, which is comparatively more sensitive to irrelevant or redundant dimensions, or to tree ensembles, which are comparatively more tolerant of them. However, this asymmetry is the standard design in the feature-selection literature: a cheap wrapper evaluator during search paired with a broader panel for validation (10) instead of a search-time classifier expensive enough to run directly within the validation panel. Future work could probe this directly by repeating the search with a different surrogate, for example, a shallow tree, and checking whether training-fold and held-out rankings are still in agreement.

The reproduction of the same LLM-pipeline ranking (i.e., Qwen2.5-14B-Instruct ranked first, Phi-4 second, and BianCang third) across two independent evaluation stages (a fixed-fold proxy score computed before any feature selection and the full downstream pipeline after it) is, to our knowledge, the first such cross-validation of a human annotation–free LLM extraction screen for a TCM structured extraction task. This suggests that the substitution is not merely convenient but empirically sound in this setting, although a single reproduction on one dataset does not establish generality.

The fact that the TCM domain model underperformed both general-purpose models is notable and, on reflection, not entirely surprising: BianCang has roughly half the parameter count of the two general models compared here (7B vs. 14B), and its domain adaptation targets syndrome differentiation and diagnostic reasoning rather than the specific structured-extraction and instruction-following behavior this task demands. This echoes a broader pattern reported for Chinese medical LLMs on clinical-reasoning benchmarks (9), where domain-adapted models can trail general-purpose ones outside the specific knowledge–recall tasks that their adaptation targets.

We do not interpret this result as evidence that domain adaptation is unhelpful for TCM natural language processing (NLP) tasks in general because gains of TCM-GPT on TCM knowledge and diagnosis tasks (7) showed it can help; only that, for this particular extraction task and prompt design, model scale and instruction-following fidelity outweighed the domain match.

A structural property of LEAF worth stating explicitly is that both of its stages are modular, plug-and-play components rather than fixed implementation choices, and the aforementioned comparisons are direct empirical demonstrations of that modularity rather than being its incidental byproducts. Stage 1 requires only that a chosen LLM return schema-constrained JSON against the fixed 35-item symptom ontology (Section 2.4); the output is mapped into the same 78-column feature-table format (Section 2.6) regardless of which model produced it, and therefore, stage 2 never needs to know which LLM ran upstream. We exercised this directly by running three architecturally distinct LLMs, two general-purpose models, and one TCM domain–adapted model through the identical downstream pipeline with no pipeline-side modification and then repeating feature selection of NSDE on the feature table of each pipeline in Section 3.3 using the same search code. Stage 2 is modular in the same sense: any algorithm capable of optimizing a bounded feature-mask vector against the two-objective (macro-F1 and feature-ratio) fitness function of Section 2.7 can take the place of one of the four MOEAs compared here, which is what let us compare dominance-based, archive-based, swarm-based, and differential evolution–based search on identical terms without redesigning the search space representation or the fitness evaluator for each paradigm. This modularity is why the two future work directions mentioned in the following—newer MOEA variants and larger or commercial LLMs—are substitutions into an already-validated architecture instead of new architectures to be built and revalidated from scratch; the contribution by LEAF is accordingly best read as that architecture, not as a claim that these particular four algorithms or three models are uniquely suited to TCM respiratory syndrome differentiation.

The generalizability of these findings to records from other institutions is untested. All records analyzed here come from TCM-SD, and several components of LEAF are adapted to its documentation conventions. The rule-based tongue, pulse, and trigger features rely on the templated structure of the four-diagnosis field of TCM-SD, whose diagnostically useful content sits in a short tongue/pulse tail (Section 2.3); records that describe these findings in free narrative or under a different template would require the rules to be rederived. The synonym lists and the corpus-keyword component of the ontology were also drawn from this corpus, and therefore, institutional or regional differences in terminology could lower extraction recall. In addition, the 14-class label set follows the normalized syndrome vocabulary of TCM-SD, whose class distribution need not match that of another hospital. The LLM stage reads free text against an ontology with synonyms and may therefore be less sensitive to documentation style than the rules, but we have not tested this. External validation on an independent syndrome-differentiation dataset, preferably from a different institution, is needed before the selected feature subsets or the LLM pipeline ranking can be assumed to transfer.

Table 8 places LEAF alongside the AI approaches to TCM syndrome differentiation reviewed in Section 1. The comparison is qualitative because the studies differ in task and data: Yao et al. (2) classified five disease categories, Liu et al. (3) addressed lung-cancer syndromes, Gong et al. (4) predicted co-occurring syndromes from structured inputs, the baselines in the study of Ren et al. (1) covered all 148 syndromes of TCM-SD, and TCMEval-SDT (5) is a 300-case evaluation benchmark, and therefore, none of their reported scores is comparable with macro-F1 on our 14-class respiratory subset. Among these approaches, LEAF is the only one that derives an explicit symptom layer from free text via ontology-constrained LLM extraction and returns a Pareto front of inspectable symptom subsets; the price of this design is the modest absolute macro-F1 reported previously in this study. Measuring that price directly, by retraining an end-to-end text classifier such as the TCM-SD baselines on the same respiratory subset and data split, is left to future work.

**Table 8:** Qualitative comparison of LEAF with the AI approaches to TCM syndrome differentiation reviewed in Section 1; differences in task and data preclude a direct numerical comparison.

Study	Input	Intermediate representation and selection	Task	Symptom-level interpretability
Yao et al. (2)	Free-text clinical records, character-level input	None; a BERT model with continued pretraining on a TCM corpus classifies the text directly	Five disease categories	No symptom-level representation
Liu et al. (3)	Free-text clinical records	BERT–CRF entity representation feeding an ensemble of deep text classifiers (RCNN, Text-HAN)	Lung cancer syndrome differentiation	No inspectable intermediate representation
Ren et al. (1), TCM-SD baselines	Free-text clinical records	None; text-classification baselines, including the domain-pretrained ZY-BERT	148 syndromes, full TCM-SD	No symptom-level representation
Gong et al. (4)	Structured inputs already present in the corpus	Features selected by single-objective multilabel ReliefF (PCC-MLRF), classified by a multilabel deep forest	Co-occurring syndromes under small samples	Selected features can be inspected
Wang et al. (5), TCMEval-SDT	300 expert-curated cases	Expert-annotated four-step diagnostic-thought chain	Evaluation benchmark for syndrome-differentiation reasoning	Reasoning chain annotated by experts
LEAF (this study)	Free-text chief complaint and history plus the four-diagnosis field	35 ontology-constrained LLM-extracted symptoms and 43 rule-based tongue, pulse, and trigger features; multiobjective evolutionary feature selection	14 respiratory syndromes, TCM-SD subset	Pareto front of inspectable symptom subsets checked against the TCM-SD knowledge base

### 4.1 Limitations

Several limitations follow directly from decisions made during the study and should guide how the results are read. First, and most consequentially, no TCM-qualified clinician reviewed the symptom ontology, the few-shot examples, or any extraction output; every knowledge source is a static, publicly available document, and the clinical accuracy and completeness of the ontology have not been verified by a practitioner. Second, without a human-annotated symptom-level gold standard, we cannot report extraction precision/recall in the conventional sense; the downstream proxy evaluation is a substitute, which was validated only by the single ranking reproduction result in Section 3.3. This proxy shows only that the feature table of a pipeline, taken as a whole, carries information about the syndrome label. It cannot confirm that any individual symptom judgment is correct, nor that a symptom recorded as present is clinically significant for the patient, and systematic extraction errors that happen to correlate with the syndrome label would go unnoticed. Therefore, the extracted symptoms should not be read as clinically validated findings. Third, rare syndromes of the respiratory subset are represented by very few held-out examples, with the smallest classes having on the order of two examples per crossvalidation fold, which limits the statistical power of any per-class analysis and contributes to the wide variance in fold-level macro-F1. Section 3.6 examines class-imbalance handling only at the classifier-training stage; imbalance-aware fitness functions or resampling inside the evolutionary search itself, including oversampling schemes that adapt SMOTE variants (42) to binary features, were not explored and are a natural next step. Fourth, each evolutionary algorithm was run for 10 seeds at a population of 100 for 100 generations. We compared the four algorithms with seed-blocked Friedman tests on training-fold macro-F1, held-out macro-F1, hypervolume, and IGD, and because held-out macro-F1 is one of the two metrics that is used to identify NSDE as the primary algorithm and the held-out score of NSDE is then reported as its headline result, this reuse carries a mild optimistic selection bias toward whichever algorithm happens to look best on the held-out set among the four candidates, distinct from classical test-set leakage as no parameters are fit against held-out labels, but a bias in the same direction. With only 10 seeds, these tests also have limited power to detect anything short of a large effect, as the non-significant training-fold and held-out comparisons illustrate, and a larger seed budget or generation count was not attempted. The internal parameters of the four algorithms were also left at their pymoo defaults, and their sensitivity was not analyzed; the four algorithms that were compared spanned only established, foundational search paradigms rather than their more recent state-of-the-art refinements, a gap discussed further under the Future Work section below. Fifth, the knowledge base–consistency metric is a coarse substring-attestation check against sources the ontology itself was built from, its ceiling was 91.4% instead of 100% since three ontology terms were attested only in the cough guideline, and the single median-seed representative solution of NSDE, which happened to reach 100%, is not representative of its typical behavior, which averaged 0.955 ± 0.039 across solutions of all 10 seeds; none of this should be read as independent clinical validation. Finally, lung abscess, feiyong, which is part of the originally intended disease scope, is entirely absent from TCM-SD and could not be studied here.

### 4.2 Future work

The four algorithms compared in this study (NSGA-II, SPEA2, CMOPSO, and NSDE) spanned the dominance-based, archive-based elitist, swarm-based, and differential evolution–based paradigms surveyed previously in this study, but each is a foundational instance of its paradigm rather than being a more recent, paradigm-specific refinement.

The literature reviewed above includes several such refinements that were not evaluated here: a self-adaptive particle swarm variant for large-scale feature selection (15); a four-subpopulation differential-evolution method targeting the paradigm of NSDE (16); an enhanced NSGA-II with improved initialization, selection, and a greedy repair strategy (17); a ranking-based preshrinking strategy for large-scale search (14); and an evolutionary-multitasking reformulation of the search itself (18); a decomposition-based algorithm such as MOEA/D (43) represents a fifth paradigm entirely absent from our comparison.

Benchmarking one or more of these more recent, state-of-the-art algorithms against the four foundational paradigms compared here is a natural next step: doing so would help clarify whether the modest, non-significant differences observed among NSGA-II, SPEA2, CMOPSO, and NSDE in this study reflect a ceiling specific to this dataset and feature space or whether more recent search refinements can meaningfully move the outcome metrics.

A second, orthogonal direction concerns the extraction stage itself. The three LLMs compared here (Qwen2.5-14B-Instruct, Phi-4, and BianCang–Qwen2.5-7B-Instruct) were all locally deployed, open-weight models in the 7–14 billion parameter range, which kept inference cost fixed and reproducible across the thousands of records processed in this study. Larger open-weight models and commercial application programming interface (API)–served models (for example, the GPT, Claude, and Gemini families) were not evaluated, and it remains an open question whether the extraction-pipeline ranking observed here, along with the modest absolute macro-F1 values that follow from it, would hold at greater model scale. Substituting a commercial API would also trade the fixed, reproducible, locally controlled inference of this study (21, 22) for per-call pricing and dependence on a third-party service; this cost would be worth weighing against any extraction-quality gain such models might offer.

Three further directions follow from the aforementioned limitations. First, the extraction stage should be validated clinically: a stratified sample of held-out records, annotated independently by two licensed TCM practitioners against the 35-item ontology, would allow per-symptom precision, recall, and interannotator agreement to be reported, and the same practitioners could review the ontology itself. Second, a parameter-sensitivity analysis, for example, over population size at a fixed evaluation budget and over variation operators of each algorithm would show whether the nominal ordering among the four algorithms is stable under tuning, and imbalance-aware fitness functions or resampling inside the search could be compared with the classifier-stage handling of Section 3.6. Third, external validation on an independent dataset from a different institution, together with end-to-end syndrome differentiation models such as the TCM-SD baselines retrained on the same respiratory subset, would show how well LEAF transfers and how much accuracy its interpretable intermediate representation costs.

## 5 Conclusion

Herein, we presented LEAF, a two-stage pipeline that extracts structured symptom features from free-text TCM respiratory disease records using locally deployed LLMs and selects interpretable feature subsets using MOEAs.

Across 10 seeds, four MOEAs spanning distinct search paradigms reduced the candidate feature set by 48%–60% on average; the mean held-out macro-F1 of every algorithm fell slightly below the full-feature baseline on point estimates, by 0.033–0.043, although a four-classifier significance test detected no significant difference between any algorithm and either the full-feature baseline or a random-feature control. NSDE emerged as the best-performing algorithm on the two outcome-relevant metrics (training-fold and held-out macro-F1, both averaged over all 10 seeds), both nominally favoring NSDE despite neither reaching significance, while a third metric, hypervolume (corroborated by IGD), instead favored NSGA-II, the algorithm with the worst mean held-out macro-F1 of the four. We treat this disagreement between the search-process quality and classification outcome as a substantive finding in its own right; we prioritize the outcome metrics in this comparison rather than retroactively picking whichever algorithm performed best on a single held-out evaluation.

The LLM pipeline ranking obtained from a human annotation–free proxy screen was reproduced after the complete feature-selection pipeline, supporting the proxy approach as a practical substitute for gold-standard annotation when clinical expert time is unavailable.

Moreover, future work should prioritize the aforementioned limitations: an eventual clinician review of the ontology and a small hand–annotated validation set reporting per-symptom precision and recall, evolutionary runs with a larger seed or generation budget and a parameter-sensitivity analysis, a decomposition-based algorithm such as MOEA/D to broaden paradigm coverage, external validation on an independent TCM syndrome differentiation dataset together with a quantitative comparison against end-to-end models, and a more principled, class-aware formulation of the knowledge-consistency objective as a live optimization target rather than as * post-hoc* check.

## Data Availability

The original data analyzed in this study are publicly available: TCM-SD can be found at https://github.com/Borororo/ZY-BERT (MIT license for the code, CC BY-NC-SA 4.0 for the data). The respiratory-disease subset construction scripts, symptom ontology, extraction prompts, feature tables, and feature-selection and validation code produced in this study are available at https://zenodo.org/records/22994254.

## Supplementary material

SUPPLEMENTARY DATA SHEET 1 Symptom ontology source traceability (S1), full LLM extraction prompt (S2), description of the feature tables (S3), and NSDE per-feature selection frequency across 10 seeds (S4). The six CSV feature tables described in S3 are available at https://doi.org/10.5281/zenodo.22994254 (data/feature_tables).
