# Supplementary material for: LEAF: benchmarking multiobjective evolutionary feature selection over large language model–extracted symptoms for interpretable Traditional Chinese Medicine respiratory syndrome differentiation
Source: Front Med (Lausanne). 2026 Oct 28;13:1990580. doi: 10.3389/fmed.2026.1990580 (PMC13752124; doi:10.3389/fmed.2026.1990580)
Supplement: Supplementary file 1 [file Datasheet1.pdf]

Supplementary Material

LEAF: Benchmarking Multi-Objective Evolutionary Feature Selection over LLM-Extracted Symptoms for Interpretable TCM Respiratory Syndrome Differentiation

Xuanhang Luo, Huan Ding, Xize Zhang, Binhuan Miao, and Li Shi

S1. Symptom Ontology Source Traceability

The table below lists all 35 symptom terms in the checklist described in Section 2.3 of the main text, together with the source, or sources, each term is traceable to: the TCM-SD knowledge base (232 candidate phrases drawn from the *Typical_performance* field of the 30 respiratory syndromes), the 2021 Expert Consensus on TCM Diagnosis and Treatment of Cough (China Association of Chinese Medicine, 2021), or unsupervised keyword extraction over the training dataset's free text. Four terms, marked "Cough guideline only" or jointly with the TCM-SD knowledge base, were added directly from the guideline; one of them, nasal flaring, is also attested in the TCM-SD knowledge base, and the other three are the three terms found unattested against the TCM-SD knowledge base in the knowledge-consistency check reported in Section 3.5 of the main text.

Table 1: The 35-item symptom checklist and the source(s) each term is traceable to.

No.	Symptom (English)	Symptom (Chinese)	Source(s)
1	Cough	咳嗽	TCM-SD knowledge base; corpus
2	Expectoration	咳痰	TCM-SD knowledge base; corpus
3	Sputum volume	痰量	TCM-SD knowledge base
4	Sputum consistency	痰质	TCM-SD knowledge base; corpus
5	Sputum color	痰色	TCM-SD knowledge base; corpus
6	Hemoptysis	咳血/咯血	TCM-SD knowledge base; corpus

No.	Symptom (English)	Symptom (Chinese)	Source(s)
7	Panting/dyspnea	气喘	TCM-SD knowledge base; corpus
8	Difficulty breathing	呼吸困难	TCM-SD knowledge base
9	Orthopnea	不能平卧	TCM-SD knowledge base
10	Wheeze sound	喘鸣	TCM-SD knowledge base; corpus
11	Nasal flaring	鼻翼煽动	TCM-SD knowledge base; Cough guideline
12	Mouth-open shoulder-raising	张口抬肩	Cough guideline only
13	Hoarse wheeze sound	喉中哮鸣有声	Cough guideline only
14	Rapid onset and resolution	发作与缓解均迅速	Cough guideline only
15	Chest tightness	胸闷	TCM-SD knowledge base; corpus
16	Chest pain	胸痛	TCM-SD knowledge base; corpus
17	Fever	发热	TCM-SD knowledge base; corpus
18	Aversion to cold	恶寒	TCM-SD knowledge base; corpus
19	Abnormal sweating	汗出异常	TCM-SD knowledge base
20	Nasal congestion and discharge	鼻塞流涕	TCM-SD knowledge base
21	Throat symptoms	咽喉症状	TCM-SD knowledge base
22	Headache and body ache	头身痛	TCM-SD knowledge base
23	Susceptibility to catching cold	易感冒	TCM-SD knowledge base
24	Fatigue	神疲乏力	TCM-SD knowledge base
25	Palpitations	心悸	TCM-SD knowledge base
26	Dizziness	头晕	TCM-SD knowledge base
27	Nausea and vomiting	恶心呕吐	Corpus only
28	Abnormal urination/defecation	二便异常	TCM-SD knowledge base; corpus

No.	Symptom (English)	Symptom (Chinese)	Source(s)
29	Edema	水肿	TCM-SD knowledge base; corpus
30	Abnormal complexion/body build	面色/形体异常	TCM-SD knowledge base
31	Soreness and weakness of the lower back and knees	腰膝酸软	TCM-SD knowledge base
32	Tinnitus	耳鸣	TCM-SD knowledge base
33	Vexing heat in the five centers	五心烦热	TCM-SD knowledge base
34	Cold extremities	肢冷	TCM-SD knowledge base
35	Reduced appetite	食欲减退	Corpus only

Note: "Corpus" refers to unsupervised keyword extraction over the training dataset's free text via the jieba segmenter; "Cough guideline" refers to the 2021 Expert Consensus on TCM Diagnosis and Treatment of Cough (China Association of Chinese Medicine, 2021). English symptom names are our translations of the original Chinese terms; synonyms for each term are reproduced verbatim in the Chinese system prompt in Section S2 below.

S2. LLM Extraction Prompt

This section reproduces, verbatim, the prompt used for LLM-based structured symptom extraction described in Section 2.4 of the main text. The prompt is written in Chinese, the language of the underlying TCM-SD clinical records (Ren et al., 2022), since translating it would change the exact tokens the models saw; an English summary of its instructions precedes the verbatim text for readers who do not read Chinese. The same system prompt, few-shot examples, and JSON schema were sent to all three LLMs, Qwen2.5-14B-Instruct, Phi-4, and BianCang-Qwen2.5-7B-Instruct, and were not used at all for the rule-based tongue/pulse/trigger features, which are extracted separately by regex, as described in Section 2.3.

S2.1 Summary of Instructions in English

The system prompt instructs the model to act as a TCM clinical-text structuring assistant. Given a chief complaint and history-of-present-illness text, the model must judge, for every one of the 35 symptom terms in a fixed checklist, whether that symptom is present, absent, or not mentioned in the text, using only the exact standardized term names in the checklist and treating any listed synonym as equivalent to its parent term. Five rules govern the judgment:

1. Symptom names must be chosen only from the fixed 35-item checklist; the model may not invent new symptom names outside that list, and a synonym listed in parentheses after a checklist term counts as a match for that term.

2. Each symptom is judged as one of three states: present, meaning the text explicitly states the patient has the symptom or an equivalent expression; absent, meaning the text explicitly states the patient does not have it, for example an explicit negation or a normal-finding statement; or not mentioned, meaning the text neither explicitly confirms nor denies it. The model is instructed not to infer absence merely from silence, and not to force a loosely related but absent term onto a checklist item.

3. A severity field is required for every symptom. It is fixed to "not applicable" when the symptom is judged absent or not mentioned. When present, five checklist items describing a descriptive attribute, sputum quantity, sputum quality, sputum color, complexion or body-form abnormality, and urination/defecation abnormality, must have the specific attribute value from the text; all other present symptoms take the specific severity description from the text if one exists, or "present but severity unknown" otherwise.

4. Every one of the 35 checklist items must appear exactly once in the output, with no omissions and no duplicates.

5. The output must strictly follow the JSON schema in Section S2.4 below, with no extra prose, explanation, markdown code-fence markers, or comments.

S2.2 System Prompt: Verbatim Chinese Original

你是一名中医内科病历文本结构化助手。给定一段病历文本（主诉 + 现病史），请从下面给定的“症状清单”中，判断每一个症状在文本中的状态，并严格按 JSON 格式输出。

规则：

1. 只能从下面的“症状清单”中选择症状名，不能自己编造清单之外的症状名称。清单中每个症状名后括号内列出的是同义词/口语变体，文本中出现同义词也算命中该症状名。

2. 对清单中的每一个症状，判断为以下三种状态之一：

- “有”：文本中明确提到患者存在该症状（或其同义表达）。
- “无”：文本中明确提到患者不存在该症状（如“无发热”“不咳嗽”“XX 正常”等否定/正常表达）。
- “未提及”：文本中既没有明确说“有”，也没有明确说“无”。

注意：只有文本存在真正的否定/正常措辞时才判“无”；不要把“未提及”误判为“无”，也不要把语义相近但清单里没有的词强行匹配到某个症状上。

3. “severity”（程度）字段填写规则：

- 若判断为“无”或“未提及”，severity 固定填“不适用”。
- 若判断为“有”：
    - 对于“痰量”“痰质”“痰色”“面色/形体异常”“二便异常”这类描述性质地/属性的条目，severity 填写文本中给出的具体属性值。
    - 对于其余条目，若文本有明确程度描述，填写该描述；若没有明确程度描述，填“存在但程度未知”。

4. 症状清单中的每一项都必须输出一条记录，不能遗漏，也不能重复。

5. 严格按 JSON schema 输出，不要输出任何多余的文字、解释、markdown 代码块标记或注释。

症状清单（共 35 项）：1. 咳嗽（咳嗽不爽、咳声重浊、咳声低、干咳、干咳无痰、阵咳、咳嗽无力、咳嗽气喘、咳嗽气短）2. 咳痰（咯痰、咳出、易咯出、不易咯出）3. 痰量（痰多、痰少、吐白痰量多）4. 痰质（痰质稀、清稀、粘痰、痰稠）5. 痰色（色白、色黄、黄白）6. 咳血/咯血（咯血、咳血、痰中带血、咳吐脓血腥臭痰）7. 气喘（喘息、气喘息粗、动则喘甚、动则尤甚、咳喘）8. 呼吸困难（短气喘促、深吸为快、气不得以续、呼多吸少）9. 不能平卧（甚则不能平卧）10. 喘鸣（喉中痰鸣、喉间哮鸣声、胸闷喘鸣）11. 鼻翼煽动（甚则鼻翼煽动）12. 张口抬肩（无同义词）13. 喉中哮鸣有声（哮鸣有声、以哮鸣声为主）14. 发作与缓解均迅速（无同义词）15. 胸闷（胸闷喘鸣、伴有胸闷、胸部痞闷、胸脘痞闷）16. 胸痛（无同义词）17. 发热（发热轻、发热口渴、恶寒发热、微恶风寒、发热微恶风寒）18. 恶寒（微恶寒、恶寒重）19. 汗出异常（无汗、自汗、汗出、汗出恶风、稍劳汗出尤甚、盗汗、潮热盗汗）20. 鼻塞流涕（鼻塞、流清涕、流浊涕）21. 咽喉症状（喉痒、咽喉肿痛、咽喉微痛、声音嘶哑、声音不扬、语声重浊）22. 头身痛（头痛、身痛无汗、头身痛）23. 易感冒（易于感冒）24. 神疲乏力（乏力、体倦乏力、四肢乏力、身困神倦、肢重嗜卧、声低懒言）25. 心悸（眩晕心悸）26. 头晕（头晕眼花、头晕目眩）27. 恶心呕吐（呕吐、恶心、口不渴或呕恶纳呆）28. 二便异常（小便不利、小便短黄、大便秘结、大便粘腻不爽、腹胀便溏、纳呆便溏、腹泻）29. 水肿（浮肿、水肿下肢为重、重者肿至全身）30. 面色/形体异常（面色淡白、面色少华、面色晄白或灰滞、形体消瘦、两颧潮红）31. 腰膝酸软（无同义词）32. 耳鸣（无同义词）33. 五心烦热（无同义词）34. 肢冷（汗出肢冷、神倦肢冷）35. 食欲减退（食欲差、纳差、胃纳差、纳呆、饮食欠

佳、食纳差）

严格按下面的 JSON schema 输出：{”symptoms”: [{”name”: ”< 清单中的症状名 >”, ”present”: ” 有 | 无 | 未提及”, ”severity”: ”< 具体值 | 存在但程度未知 | 不适用 >”}, ...]}

S2.3 Few-Shot Examples

Four few-shot examples were appended to the system prompt as alternating user/assistant turns, in the fixed order below, for every extraction call. Each example pairs a real training-dataset case's chief complaint and history-of-present-illness text with a complete, 35-item reference answer hand-constructed by the project team against the symptom ontology, as described in Section 2.4. All four examples are reproduced verbatim; Example 1's full assistant answer is shown in full to illustrate the required output format, and Examples 2–4 are given with their user turns in full and their answers summarized to the symptoms judged present, since the not-mentioned/absent entries follow the same exhaustive 35-item pattern as Example 1.

Example 1.

User: 主诉：咳嗽咳痰伴畏寒发热 20 余天
现病史：患者 20 余天前，劳累受凉后出现乏力，全身酸痛，伴畏寒，未测体温，后出现咳嗽，咳白粘痰，不易咳出，伴轻度胸闷气喘不适，活动后加重，仍未引起重视，后出现畏寒发热，测体温 38℃，自服阿莫西林、清开灵等药物，体温峰值下降，但仍有低热，体温多在 37.5℃ 左右，每日午后发热，自服退热药物可下降至正常，伴有轻度畏寒，无明显寒颤，轻度胸闷。病程中，患者食欲差，偶有反酸不适，无胸痛，无咯血，无晕厥，食纳差，大小便正常，体重下降不明显。

Assistant, all 35 items, verbatim:

```
{"symptoms":[
{"name":"咳嗽","present":"有","severity":"存在但程度未知"},
{"name":"咳痰","present":"有","severity":"存在但程度未知"},
{"name":"痰量","present":"未提及","severity":"不适用"},
{"name":"痰质","present":"有","severity":"黏（不易咳出）"},
{"name":"痰色","present":"有","severity":"白"},
{"name":"咳血/咯血","present":"无","severity":"不适用"},
{"name":"气喘","present":"有","severity":"轻度"},
{"name":"呼吸困难","present":"未提及","severity":"不适用"},
{"name":"不能平卧","present":"未提及","severity":"不适用"},
{"name":"喘鸣","present":"未提及","severity":"不适用"},
{"name":"鼻翼煽动","present":"未提及","severity":"不适用"},
{"name":"张口抬肩","present":"未提及","severity":"不适用"},
```

```
{"name":"喉中哮鸣有声","present":"未提及","severity":"不适用"},
{"name":"发作与缓解均迅速","present":"未提及","severity":"不适用"},
{"name":"胸闷","present":"有","severity":"轻度"},
{"name":"胸痛","present":"无","severity":"不适用"},
{"name":"发热","present":"有","severity":"低热"},
{"name":"恶寒","present":"有","severity":"轻度"},
{"name":"汗出异常","present":"未提及","severity":"不适用"},
{"name":"鼻塞流涕","present":"未提及","severity":"不适用"},
{"name":"咽喉症状","present":"未提及","severity":"不适用"},
{"name":"头身痛","present":"有","severity":"存在但程度未知（全身酸痛）"},
{"name":"易感冒","present":"未提及","severity":"不适用"},
{"name":"神疲乏力","present":"有","severity":"存在但程度未知"},
{"name":"心悸","present":"未提及","severity":"不适用"},
{"name":"头晕","present":"未提及","severity":"不适用"},
{"name":"恶心呕吐","present":"未提及","severity":"不适用"},
{"name":"二便异常","present":"无","severity":"不适用"},
{"name":"水肿","present":"未提及","severity":"不适用"},
{"name":"面色/形体异常","present":"无","severity":"不适用"},
{"name":"腰膝酸软","present":"未提及","severity":"不适用"},
{"name":"耳鸣","present":"未提及","severity":"不适用"},
{"name":"五心烦热","present":"未提及","severity":"不适用"},
{"name":"肢冷","present":"未提及","severity":"不适用"},
{"name":"食欲减退","present":"有","severity":"存在但程度未知"}
]}
```

Example 2.

User: 主诉：反复咳喘 30 年，加重 1 周
现病史：患者 30 年前受凉后出现咳嗽、咳痰、喘憋，于当地医院就诊，予以对症治疗后改善。此后 10 余年间，咳嗽、咳痰及喘憋症状反复发作，多在受凉及季节交替时加重，每年发作时间超过 3 个月，诊断为慢性阻塞性肺病，予以药物化痰、止喘及消炎等对症治疗后改善。1 周前因受凉后出现气喘加重，动则尤甚，咳嗽、咳黄白粘痰，无发热，无胸痛，自服药物效果欠佳。今来我院就诊，门诊以慢性阻塞性肺疾病收入我区住院系统治疗。近 1 周来，患者神志清，精神可，咳嗽、咳痰、气喘，饮食一般，睡眠可，二便正常。

Assistant, symptoms judged present; all remaining 30 checklist items were judged not mentioned or absent following Example 1's pattern:

咳嗽 – 存在但程度未知; 咳痰 – 存在但程度未知; 痰质 – 黏; 痰色 – 黄白; 气喘 – 动则尤甚 （发热、胸痛、二便异常均判定为“无”） 。

Example 3.

User: 主诉：反复咳喘 3 年余, 加重 2 周
现病史：患者 3 年余前无明显诱因出现咳嗽、咳痰，干咳为主, 外院就诊，诊断为肺纤维化。未予特殊治疗。2 周前患者因受凉后出咳喘加重，动则尤甚，咳嗽、咳中等量黄白粘痰，无发热，无明显胸痛，未予重视及治疗，今为求进一步诊治，今来我院就诊。患者病程中，无恶寒发热，无恶心呕吐，无咳血胸痛，双下肢稍有水肿，胃纳差，睡眠一般，小便量少，大便正常。

Assistant, symptoms judged present; cough-blood, chest pain, fever, chills, and nausea/vomiting explicitly judged absent, remainder not mentioned:

咳嗽 – 存在但程度未知; 咳痰 – 中度; 痰量 – 中等; 痰质 – 黏; 痰色 – 黄白; 气喘 – 动则尤甚; 二便异常 – 小便量少; 水肿 – 轻度（双下肢稍有水肿）; 食欲减退 – 存在但程度未知（胃纳差）。

Example 4.

User: 主诉：咳嗽、咳痰 1 月余
现病史：患者 1 月前受凉后出现咳嗽，咳痰，痰少，色黄，易咳出，无明显胸闷憋喘。在当地医院给予头孢、左氧氟沙星抗炎等对症治疗后，症状未见明显好转，仍咳嗽、咳痰。为求进一步诊治，今来我院门诊，收入我区。病程中，无胸痛，无恶心呕吐，无腹痛腹泻，饮食可，夜间睡眠可，二便正常。

Assistant, symptoms judged present; wheeze/dyspnea, chest tightness, chest pain, nausea/vomiting, urination/defecation abnormality, and reduced appetite explicitly judged absent, remainder not mentioned:

咳嗽 – 存在但程度未知; 咳痰 – 易咳出; 痰量 – 少; 痰色 – 黄。

S2.4 JSON Output Schema

The following JSON Schema was passed to vLLM's `guided_json` constrained-decoding parameter for every extraction call, with `<35 checklist names>` expanded to the exact 35 standardized symptom names enumerated in Section S2.2 above; the three enumerated values of `present` are the literal Chinese tokens defined in the system prompt's rule 2, meaning present, absent, and not-mentioned respectively.

```
{
"type": "object",
"properties": {
"symptoms": {
"type": "array",
```

```
"minItems": 35,
"maxItems": 35,
"items": {
"type": "object",
"properties": {
"name": {"type": "string", "enum": [<35 checklist names>]},
"present": {"type": "string", "enum": ["有", "无", "未提及"]},
"severity": {"type": "string"}
},
"required": ["name", "present", "severity"]
}
}
},
"required": ["symptoms"]
}
```

S3. Feature Tables

This item provides the full case-by-feature tables described in Section 2.6 of the main text, as six comma-separated-value (CSV) files, one per LLM pipeline and data split:

- `train_qwen25-14b.csv` (2596 rows) and `heldout_qwen25-14b.csv` (650 rows), for the Qwen2.5-14B-Instruct pipeline;
- `train_phi-4.csv` (2601 rows) and `heldout_phi-4.csv` (651 rows), for the Phi-4 pipeline;
- `train_biancang-7b.csv` (2505 rows) and `heldout_biancang-7b.csv` (625 rows), for the BianCang-Qwen2.5-7B-Instruct pipeline.

Each row is one clinical record; row counts fall short of the full training-dataset (2602) and held-out (651) totals reported in Section 2.2 of the main text wherever that pipeline's LLM did not return valid JSON for a given record, consistent with the 96.0–100% valid-JSON coverage reported in Section 2.4.

Each file has 81 columns: three metadata columns, `user_id` (the record identifier), `syndrome_merged` (the 14-class syndrome label described in Section 2.1), and `cv_fold` (the 5-fold cross-validation assignment described in Section 2.2, populated only in the training-dataset files and left empty in the held-out files); 35 LLM-derived symptom columns, named `sym_<term>` for each of the 35 checklist items listed in Supplementary Material S1, coded 1 if the LLM judged the symptom present and 0 if absent or not mentioned; and 43 rule-derived columns, named `rule_<feature>`, for the regex-extracted tongue-color, coating-color, coating-quality, pulse, and trigger features described in Section 2.3, coded 1 if the feature was detected, in the record's four-diagnosis field

for tongue, coating, and pulse features and anywhere in the record text for trigger features, and 0 otherwise. Table 2 in the main text reproduces a six-row excerpt of `train_qwen25-14b.csv`.

S4. NSDE Feature-Selection Stability Across Ten Seeds

This item provides the full per-feature counts underlying the stability analysis in Section 3.4 of the main text. Across ten random seeds, NSDE's best-macro-F1 Pareto solution on the Qwen2.5-14B-Instruct pipeline selected 76 of the 78 candidate features at least once; the table below lists all 76, together with the number of seeds, out of ten, in which each was selected, sorted from most to least stable. The 20 features selected in all ten seeds reproduce Table 5 of the main text; the remaining two candidate features, both rule-derived, were never selected in any of the ten seeds and do not appear below.

Table 2: Selection frequency, out of ten seeds, for all 76 features selected by NSDE's best-macro-F1 Pareto solution at least once.

**Feature**	**Category**	**Selected**
Coating color: white	Rule (coating color)	10/10
Coating color: yellow	Rule (coating color)	10/10
Coating quality: thin	Rule (coating quality)	10/10
Pulse: deep	Rule (pulse)	10/10
Pulse: rapid	Rule (pulse)	10/10
Pulse: thready	Rule (pulse)	10/10
Pulse: weak	Rule (pulse)	10/10
Pulse: wiry	Rule (pulse)	10/10
Tongue color: purple	Rule (tongue)	10/10
Trigger: cold exposure	Rule (trigger)	10/10
Trigger: seasonal change	Rule (trigger)	10/10
Abnormal urination/defecation	Symptom	10/10
Difficulty breathing	Symptom	10/10
Edema	Symptom	10/10
Fatigue	Symptom	10/10
Orthopnea	Symptom	10/10
Panting/dyspnea	Symptom	10/10
Reduced appetite	Symptom	10/10
Sputum color	Symptom	10/10
Sputum consistency	Symptom	10/10
Coating quality: thick	Rule (coating quality)	9/10
Tongue color: pale	Rule (tongue)	9/10

**Feature**	**Category**	**Selected**
Tongue color: pale-red	Rule (tongue)	9/10
Chest tightness	Symptom	9/10
Cough	Symptom	9/10
Nausea and vomiting	Symptom	9/10
Pulse: floating	Rule (pulse)	8/10
Pulse: slippery	Rule (pulse)	8/10
Pulse: tight	Rule (pulse)	7/10
Fever	Symptom	7/10
Coating quality: greasy	Rule (coating quality)	6/10
Coating quality: slippery	Rule (coating quality)	6/10
Tongue color: red	Rule (tongue)	6/10
Expectoration	Symptom	6/10
Hemoptysis	Symptom	6/10
Coating color: black	Rule (coating color)	5/10
Pulse: moderate	Rule (pulse)	4/10
Pulse: scattered	Rule (pulse)	4/10
Chest pain	Symptom	4/10
Nasal flaring	Symptom	4/10
Wheeze sound	Symptom	4/10
Coating color: gray	Rule (coating color)	3/10
Coating quality: dry	Rule (coating quality)	3/10
Coating quality: no coating	Rule (coating quality)	3/10
Pulse: choppy	Rule (pulse)	3/10
Pulse: hurried	Rule (pulse)	3/10
Pulse: large	Rule (pulse)	3/10
Tongue color: bluish-purple	Rule (tongue)	3/10
Hoarse wheeze sound	Symptom	3/10
Mouth-open shoulder-raising	Symptom	3/10
Rapid onset and resolution	Symptom	3/10
Throat symptoms	Symptom	3/10
Pulse: excess	Rule (pulse)	2/10
Pulse: faint	Rule (pulse)	2/10
Tongue color: dark	Rule (tongue)	2/10
Trigger: overexertion	Rule (trigger)	2/10
Abnormal complexion/body build	Symptom	2/10
Cold extremities	Symptom	2/10
Dizziness	Symptom	2/10
Headache and body ache	Symptom	2/10

**Feature**	**Category**	**Selected**
Palpitations	Symptom	2/10
Sputum volume	Symptom	2/10
Susceptibility to catching cold	Symptom	2/10
Tinnitus	Symptom	2/10
Vexing heat in the five centers	Symptom	2/10
Coating quality: scanty coating	Rule (coating quality)	1/10
Pulse: deficient	Rule (pulse)	1/10
Pulse: intermittent	Rule (pulse)	1/10
Pulse: knotted	Rule (pulse)	1/10
Tongue color: crimson	Rule (tongue)	1/10
Tongue color: pale-purple	Rule (tongue)	1/10
Tongue color: pale-white	Rule (tongue)	1/10
Abnormal sweating	Symptom	1/10
Aversion to cold	Symptom	1/10
Nasal congestion and discharge	Symptom	1/10
Soreness and weakness of the lower back and knees	Symptom	1/10
